# Metabolic Footprints of *Burkholderia* Sensu Lato Rhizosphere Bacteria Active against Maize *Fusarium* Pathogens †

**DOI:** 10.3390/microorganisms9102061

**Published:** 2021-09-29

**Authors:** Guadalupe C. Barrera-Galicia, Héctor A. Peniche-Pavía, Juan José Peña-Cabriales, Sergio A. Covarrubias, José A. Vera-Núñez, John P. Délano-Frier

**Affiliations:** 1 Centro de Investigación y de Estudios Avanzados del Instituto Politécnico Nacional, Unidad Irapuato, Irapuato 36824, Guanajuato, Mexico; guadalupe.barrera@cinvestav.mx (G.C.B.-G.); hector.peniche@cinvestav.mx (H.A.P.-P.); juan.pena@cinvestav.mx (J.J.P.-C.); 2 Área de Ciencias de la Salud, Ciudad Universitaria Campus Siglo XXI, Universidad Autónoma de Zacatecas, Zacatecas 98160, Zacatecas, Mexico; sergio.hernandez@uaz.edu.mx (S.A.C.); jose.vera@cinvestav.mx (J.A.V.-N.)

**Keywords:** biocontrol, *Burkholderia*, *Paraburkholderia*, DIESI-MS, *Fusarium*, siderophores

## Abstract

Consistent with their reported abundance in soils, several *Burkholderia* sensu lato strains were isolated from the rhizosphere of maize plants cultivated at different sites in central México. Comparative analysis of their 16S rRNA gene sequences permitted their separation into three distinctive clades, which were further subdivided into six other clusters by their close resemblance to (1) *Trinickia dinghuensis*; (2) *Paraburkholderia kirstenboschensis*, *P. graminis*, *P. dilworthii* and *P. rhynchosiae*; (3) *B. gladioli*; (4) *B. arboris*; (5) *B. contaminans*, or (6) *B. metallica* representative species. Direct confrontation assays revealed that these strains inhibited the growth of pathogenic *Fusarium oxysporum* f. sp. *radicis-lycopersici*, and *F. verticillioides* within a roughly 3–55% inhibition range. The use of a DIESI-based non-targeted mass spectroscopy experimental strategy further indicated that this method is an option for rapid determination of the pathogen inhibitory capacity of *Burkholderia* sensu lato strains based solely on the analysis of their exometabolome. Furthermore, it showed that the highest anti-fungal activity observed in *B. contaminans* and *B. arboris* was associated with a distinctive abundance of certain *m*/*z* ions, some of which were identified as components of the ornbactin and pyochelin siderophores. These results highlight the chemical diversity of *Burkholderia* sensu lato bacteria and suggest that their capacity to inhibit the *Fusarium*-related infection of maize in suppressive soils is associated with siderophore synthesis.

## 1. Introduction

Maize is extensively cultivated in the world, with a production rate of about 990.6 Mt/year, ranking as the third most important cereal for human consumption. During its vegetative cycle, the production of maize is conditioned by various biotic and abiotic factors. *Fusarium* spp. is a ubiquitous soil-borne plant pathogenic fungus that can cause highly damaging infections characterized by severe decay of seeds, roots, stems, ears or kernels [1,2]. *Fusarium verticillioides* (*Gibberella fujikuroi* mating population A; teleomorph, *Gibberella moniliformis* Wineland) is the prevalent species of *Fusarium* in distinct landscapes [3,4], and the losses caused by this fungus can vary from 7% to 38% [5].

A significant diversity of both deleterious and beneficial microbial species lives in association with the rhizosphere of most agricultural crops. Certain bacterial components of this root microbial community, known as plant growth promoting rhizobacteria (PGPR), are known to enhance plant growth and fitness [6]. Plant growth promotion can take place indirectly when PGPR prevent or diminish the damage caused by pathogenic microorganisms, an effect frequently produced through the production of secondary metabolites with strong inhibitory activity [7,8]. Examples of bacteria having this capability are found among species of the genera *Serratia*, *Bacillus*, *Brevibacillus*, *Lysobacter*, *Pseudomonas* and *Burkholderia* [9,10,11].

The genus *Burkholderia* belongs to a subphylum of the β-proteobacteria. It currently involves around 100 species with widespread distribution in frequently contrasting ecological niches [12]. The versatile lifestyles and the capability to develop in numerous habitats that characterize *Burkholderia* species are mainly due to their genomic and metabolic plasticity [13]. Several species of this genus have plant growth promoting activity, including several from the so-called *Burkholderia cepacia* complex (Bcc). Beneficial interactions with plants reported for *Burkholderia* species include nitrogen fixation, 1-aminocyclopropane-1-carboxylate (ACC) deaminase enzyme activity, phosphate solubilization and biological control of pathogens through the synthesis of siderophores, organic volatile compounds, antibiotics or lipopeptides [14,15,16,17,18,19].

Bcc members and other *Burkholderia* species, such as *B. tropica*, *B. silvatlantica*, *B. sartisoli*, *B. graminis*, *B. phytofirmans*, *B. terrestris*, *B. telluris* and *B. unamae*, represent a significant percentage of the total cultivable bacteria living within the rhizosphere of plants [20]. In this respect, various studies have reported that several species of the Bcc constitute an important segment of the cultivable root microbial communities found in the rhizosphere of plants belonging to the Gramineae family. For example, in the maize rhizosphere, *B. cenocepacia*, *B. cepacia*, *B. multivorans*, *B. ambifaria* and *B. pyrrocinia* represent between 0.5% and 6% of the total bacterial population [7,20,21].

Since its initial proposal in 1992, the taxonomic classification of *Burkholderia* has been constantly changing. This continuous flux has led to the suggestion of splitting this genus into two phylogenetic clusters [12,22]. Following this concept, the first clade should include only bacteria reported as plant or animal pathogens, such as *B. rhizoxinica*, *B. gladioli*, *B. mallei* and *B. pseudomallei*, along with the 20 species of the Bcc associated with serious respiratory infections in humans [20,23,24]. Other bacteria isolated from fungi or environmental samples (e.g., soil and water), as well as endophytic and free-living rhizosphere strains able to establish beneficial interactions with plants, should be incorporated into the second taxonomic clade [13,25,26]. The last clade has been accepted by the International Committee on Systematics of Prokaryotes as a new genus denominated *Paraburkholderia*, which includes several species having a potential biotechnological use in agriculture. This designation, however, remains controversial [12,22,27,28].

Current molecular classification methods allow an accurate classification of different environmental *Burkholderia* strains. Nevertheless, novel approaches in microbial characterization may help explore the largely unexplored exometabolome of these important rhizosphere microorganisms, which are known to secrete a wide array of secondary metabolites (e.g., cepalycin, pyrrolnitrin, components of the xylocandin complex, occidiofungin and ornibactin) that are active against soil-borne fungal plant pathogens [29,30,31]. In this respect, metabolic foot-printing was developed as a convenient, reproducible and high-throughput technique for the physiological-level characterization of microorganisms [32]. Recently, the use of untargeted mass spectrometry methods, particularly direct liquid injection electrospray ionization (DIESI-MS), for the study of plant–microbe interactions has greatly increased in the past few years [33]. In this context, the present study describes a metabolic foot-printing approach to explore the exometabolome secreted by different *Burkholderia* sensu lato strains isolated from the rhizosphere of maize plants. Its main objectives were to develop a rapid, economical and accurate methodology to determine the chemical basis of the differential anti-fungal activity observed in *Burkholderia* environmental strains isolated from maize plant rhizospheres.

## 2. Materials and Methods

### 2.1. Soil Samples

A collection of bacteria of the genus *Burkholderia* was generated from the rhizosphere of maize plants. They were isolated from 60 different soil samples obtained from 4 maize cultivation sites located in the state of Guanajuato, Mexico (coordinates: 20°38′19.2″ N 101°28′06.6″ W; 20°45′05.6″ N 101°23′30.6″ W; 20°39′21.6″ N 101°24′ 13.2″ W and 20°32′08.8″ N 101°32′53.2″ W). Thus, in each site, representative samples of the rhizosphere were taken from three neighboring plants situated at five randomly distributed points in the cultivation area. The samples were transported in refrigerated boxes lined with cooling gel packs and were immediately stored at 4 °C upon arrival to the laboratory.

### 2.2. Bacteria Isolation

The isolation of bacteria was performed using BAz medium, which is widely used for *Burkholderia* spp. [14]. However, some modifications in the formulation were applied to expand the diversity of the isolated species. Briefly, the media contained the following components (in g/L): carbon source, 2.0; K_2_HPO_4_, 0.4; KH_2_PO_4_, 0.4; MgSO_4_·7H_2_O, 0.2; CaCl_2_, 0.02; Na_2_MoO_4_·H_2_O, 0.002; FeCl_3_, 0.01; bromothymol blue, 0.075; yeast extract, 0.5; and agar, 15.0. It was adjusted to a pH of 5.7 with KOH (50%). In this study, azelaic acid or mannitol were used as carbon sources. After autoclaving (121 °C during 20 min), 70 mg/L of cycloheximide sterilized by microfiltration (0.22 µm) was added to inhibit fungal growth.

Ten grams of soil from each sample were placed in 90 mL of a 0.85% NaCl solution and were homogenized with constant stirring at 150 rpm for 60 min. Next, the denser soil aggregates were allowed to settle for 30 min at room temperature. Serial dilutions (10^−2^; 10^−3^ and 10^−4^) of the clarified soil suspensions were prepared, and 1 mL of each dilution was spread on the BAz solid media. Petri dishes were incubated for 48 h at 28 °C. Three independent replicates of each dilution and carbon source were used. Single, morphologically distinct colonies were selected for purification. These were streak-plated repeatedly onto fresh BAz media until axenic cultures were obtained.

### 2.3. Phylogenetic Identification and Classification

To obtain genomic DNA, the selected strains were grown in a nutrient broth for 24 h at 100 rpm and 28 °C. Then, 1 mL aliquots of the cultures were centrifuged at 5000 rpm for 10 min. Genomic DNA extraction from the bacterial cell pellets was performed using the PureLink Genomic DNA Mini Kit (Invitrogen, Carlsbad, CA, USA). The amplification of 16S rRNA gene fragments was performed by PCR amplification using the bacterial universal primers 27F and 1492R. The reaction conditions were the following: an initial cycle of denaturation (95 °C/3 min), followed by 35 amplification cycles (95 °C/30 s; 48 °C/30 s and 72 °C/1.5 min) and a final elongation step at 72 °C for 2 min. The PCR products containing the amplified 16S rRNA gene fragments were purified using the PureLink Quick PCR commercial package (Invitrogen) and sent for sequencing to the National Laboratory of Genomics for Biodiversity (Langebio; Cinvestav; Advanced Genomics Unit at Irapuato, Mexico).

The sequences generated were manually edited and analyzed using the CLC Sequence Viewer 7 program (CLC Bio). To identify the bacteria, each sequence was aligned using the EzBioCloud 16S database [34]. Phylogenetic analyses were performed using the maximum likelihood method along with the MEGA-X program [35]. The “find best model” tool was used to evaluate the substitution models. The approximation analysis (or “bootstrap”) was performed with 1000 replicates.

### 2.4. Fungal Strain Culture Conditions

Two fungal strains of the genus *Fusarium* were used: (1) *Fusarium oxysporum* f. sp. *radicis-lycopersici* (ATCC 60095 collection), kindly provided by Dr. Dora Linda Guzman (Mycotoxins laboratory, Cinvestav at Irapuato, Mexico), and (2) *Fusarium verticillioides* (Saccardo) Nirenberg MF-257 (ATCC 36792 collection). A conidia suspension of each strain was prepared from mature potato dextrose agar (PDA) in 10-day cultures. Briefly, the PDA plates were covered with 5 mL of sterile 0.01% Tween 20, and the fungal colonies were gently scraped using a sterile slide. After 5 min, the suspension was transferred to a tube and centrifuged at 5000 rpm for 10 min. The supernatant was discarded, and the remaining conidia were resuspended in 10 mL of 0.01% Tween 20. Aliquots containing 1 × 10^6^ conidia/mL were prepared and stored at 4 °C.

### 2.5. In Vitro Evaluation of Antagonistic Activity

The *in vitro* antagonistic activity was assessed by the dual culture technique [36]. Thus, each soil *Burkholderia* strain isolate was confronted against the two *Fusarium* strains mentioned above. A pre-inoculum of each *Burkholderia* strain was prepared in 10 mL of nutrient broth. After 24 h of incubation (150 rpm at 28 °C), 1 mL aliquots were centrifuged at 5000 rpm for 5 min. The pellets were suspended in a sterile 0.85% NaCl solution to obtain an OD_595_ of 0.2. The bacterial inocula were always prepared fresh just before the confrontation assay. Dual culture assays were performed in 8 cm Petri dishes containing PDA. Twenty µL aliquots of bacterial inocula were streaked on the agar, and then 10 µL of the conidia suspension was inoculated on sterile filter paper discs (0.7 cm in diameter) that were placed 5 cm from the bacteria streak on the opposite side of each plate. The control treatments consisted of plates inoculated with the respective fungal pathogens only. Fungal growth inhibition was recorded as the percentage reduction of the fungal colony’s radial growth 10 days after incubation at 28 °C. Five (*F. verticillioides*) and three (*F. oxysporum*) biological replicates of fungal inhibition growth assays per bacterial isolate were performed. The antagonistic activity data was analyzed by a standard analysis of variance (ANOVA) and Tukey’s post hoc tests with a significance level of *p* ≤ 0.05. These were performed using R Studio software [37].

### 2.6. Evaluation of Plant Growth Promotion Traits

*Indoleacetic acid (IAA) production*. IAA production was evaluated in Erlenmeyer flasks containing 50 mL of nutrient broth supplemented with 100 µg/L of L-tryptophan (Sigma, St. Louis, MO, USA). Aliquots (100 µL) of bacterial inocula were incubated for 72 h (150 rpm at 28 °C). Culture supernatants were obtained by centrifugation at 10,000 rpm for 5 min. IAA quantification was performed according to the procedure of Glickmann and Dessaux [38]. Briefly, 1 mL of supernatant was mixed with 2 mL of a Salkowski reagent (FeCl_3_ 4.5 g/L in 10.8 M H_2_SO_4_). The mixture was left in the dark for 30 min at room temperature, followed by measurement of the absorbance at 450 nm using a microplate spectrophotometer (TECAN GENios; Groedig, Austria). Three biological replicates per bacterial isolate were performed.

*Phosphate solubilization*. The quantitative estimation of phosphate solubilization (PS) was conducted using Erlenmeyer flasks containing 100 mL of PVK medium [39]. These were performed in triplicate with inocula of each bacterial strain. The cultures were incubated in a rotatory shaker (150 rpm) for 4 days at 28 °C. The cell biomass was separated by centrifugation (10,000 rpm for 15 min), and the amount of soluble phosphate in the supernatants was determined by the ascorbic acid colorimetric method at 880 nm [40]. These assays were performed in a microplate spectrophotometer (Bio-Rad, Hercules, CA, USA).

*Nitrogen fixation activity*. To determine the nitrogen fixing capability, an acetylene reduction assay (ARA) was performed essentially as described by Cavalcante and Döbereiner [41]. Briefly, 10 mL serum vials were filled with 5 mL of a semi-solid LGI medium prepared with some modifications. Its composition in g/L was as follows: K_2_HPO_4_, 0.2; KH_2_PO_4_, 0.6; MgSO_4_, 0.2; CaCl_2_, 0.02; Na_2_MoO4, 0.002; FeCl_3_, 0.01; bromothymol blue, 0.075; agar, 3.0; and sucrose, 5.0. The medium was adjusted to a pH of 6.0 with KOH. It was inoculated with 25 µL of bacterial inocula and incubated for 48 h at 28 °C. The acetylene reduction was estimated by changing the cotton plugs of the vials with rubber covers immediately after the injection of 13% *v/v* acetylene (C_2_H_2_) gas. After 24 h, the ethylene (C_2_H_4_) concentration was estimated in a gas chromatograph (YL6500-GC; Anyang, Korea) equipped with a 30 m long HP-Plot/Q column (0.53 mm internal diameter) and a flame-ionization detector. The gas chromatograph was operated at 120 °C using helium as the carrier gas. Three biological replicates of each bacterium were analyzed.

*Exopolysaccharide (EPS) production*. The Congo red agar method was used as a qualitative test to detect the capacity to produce exopolysaccharides [42,43]. A single colony of each strain was streaked on an ATCC no. 14 medium supplemented with 0.8 g/L of Congo red stain. The cultures were incubated for 48 h at 28 °C. An EPS positive result was indicated by the development of black colonies, while the colonies of non-slime producers remained pink.

### 2.7. Analysis of Extracellular Metabolite Production

*Sample preparation*. The secreted metabolites produced by the *Burkholderia* strains analyzed were obtained from bacteria cultured in M9 minimal medium. Thus, 100 µL aliquots of bacterial inocula were cultured in 150 mL of M9 medium containing the following in g/L: Na_2_HPO_4_, 6.0; KH_2_PO_4_, 3.0; NaCl, 0.5; NH_4_Cl, 1.0; MgSO_4_, 0.2; CaCl_2_, 0.01; and glucose, 2.0. The medium was adjusted to a pH of 7.4 with 50% KOH. After 48 h, with stirring at 150 rpm and at 28 °C, the cultures were centrifuged at 10,000 rpm for 30 min. The cell-free supernatants were filter-sterilized twice to remove bacterial biomass, first with a Nalgene 0.45-µm nylon syringe filter and then using a 0.22-µm polyvinylidene fluoride membranes (Millex GV; Carrigwohill, Cork, Ireland). The controls consisted of uninoculated M9 medium.

Solid phase separation (SPE) using 300 mg Maxi-Clean Sep-Pak C18 sorbent cartridges (Waters Corporation; Milford, MA, USA) was used as the sample preparation method. The cartridges were conditioned at a flow rate of 1 mL/min with 5 mL of methanol and 5 mL of ultra-pure water. The samples (100 mL) were loaded into the cartridges using a vacuum to achieve a flow rate of 10 mL/min. The sample concentration was achieved by eluting the material held by the SPE cartridges with 1 mL of 50% chromatography-grade methanol (LiChrosolv; Merck, Darmstadt, Germany). Prior to DIESI-MS analysis, the metabolites were filtered through 0.22 µm pore size Captiva Econofilter polytetrafluoroethylene membranes (Agilent Technologies Inc.; Santa Clara, CA, USA), and the samples were stored at −60 °C until used. Biological replicas were obtained from three independent bacterial cultures and extractions.

*Untargeted DIESI-MS analysis*. DIESI-MS was used as previously described [44,45]. The samples were injected directly with a constant 10 μL/min flow rate with a syringe pump loaded with 100 μL of the sample extract. The settings for the SQ2 equipment (Waters) were the following: 3 kV voltage for the capillary and 30 V for the cone and temperatures set to 100 °C at the source and 250 °C for the solvation gas. The desolvation gas flow was 250 L/h and 50 L/h in the cone. The mass software implemented for data acquisition was MassLynx™ 4.1 software (Waters). Continuous spectra were collected in a range of 0–800 *m*/*z* in positive ion mode only. Each reading lasted 5 min and always included at least six technical replicates of three biological replicates per bacterial cell-free extract. Due to ion suppression effects, no collection of data in the negative ionization mode was possible.

*MS data treatment and evaluation*. MS raw data preprocessing for peak alignment and binning was performed with R statistical software [46] using ChemometricsWithR [47] and previously reported scripts [44,48]. MetaboAnalyst 5.0 [49] was implemented for data transformation and multivariate statistical analyses. The MS data were scaled using the autoscaling function (mean-centered and divided by the standard deviation of each variable). One-way ANOVA was executed with a Tukey’s post hoc test and a *p*-value threshold of 0.001 to assign statistical significance to the difference in ion intensity signals detected in the profiles produced by each of the strains studied. Multivariate unsupervised and supervised methods such as principal component analysis (PCA), random forest (RF) and hierarchical cluster analysis were employed to group the *Burkholderia* strains according to their exometabolome footprints.

*MS/MS method*. XEVO TQD equipment (Waters) was used for the MS/MS analysis, which was set to the following parameters: a 3 kV capillary voltage and 30 V for the cone, 150 °C source temperature and 250 °C for the desolvation gas. The desolvation gas flow rate was 250 L/h and reduced to 50 L/h at the cone. The samples were injected directly with a constant 10 μL/min flow rate. MS fragmentation allowed the identification of metabolites by employing the Daughters method of Masslynx 4.1 as described by Peniche-Pavía and Tiessen [50]. Six energy levels were applied: 10, 20, 40, 50, 60 and 70 V. Parent ion selection was performed based on ions with a significant difference in ANOVA MS analysis and included differential (highest mean decrease in accuracy value) ions obtained in the RF tests. A commercial standard of pyochelin (Toronto Research Chemicals, Toronto, ON, Canada), a known *Burkholderia* siderophore, was used for identification purposes.

## 3. Results

### 3.1. Identification and Classification of the Isolated Rhizobacteria

Twelve *Burkholderia* and *Paraburkholderia* strains were isolated from the soil samples using the semi-selective BAz medium. High sequence similarities (>97%) with the 16S rRNA genes compiled in the EzBioCloud 16S database permitted the identification of the *Burkholderia* sensu lato strains listed in Table S1. Ten different species were identified: *Burkholderia contaminans* MSR2; *B. arboris* 1Ac4; *B. metallica* 1Ac2; *B. ubonensis* PEI4; *B. gladioli* 1Ac1; *B. gladioli* 2 Ma15; *B. gladioli* 1Ma4; *Paraburkholderia kirstenboschensis* SCV25; *P. graminis* SCV16; *P. dilworthii* FCV2; *P. rhynchosiae* SCV21; and *Trinickia dinghuensis* 2Ma17 [51] basonym *P. dinghuensis* [52].

**Figure 1:**
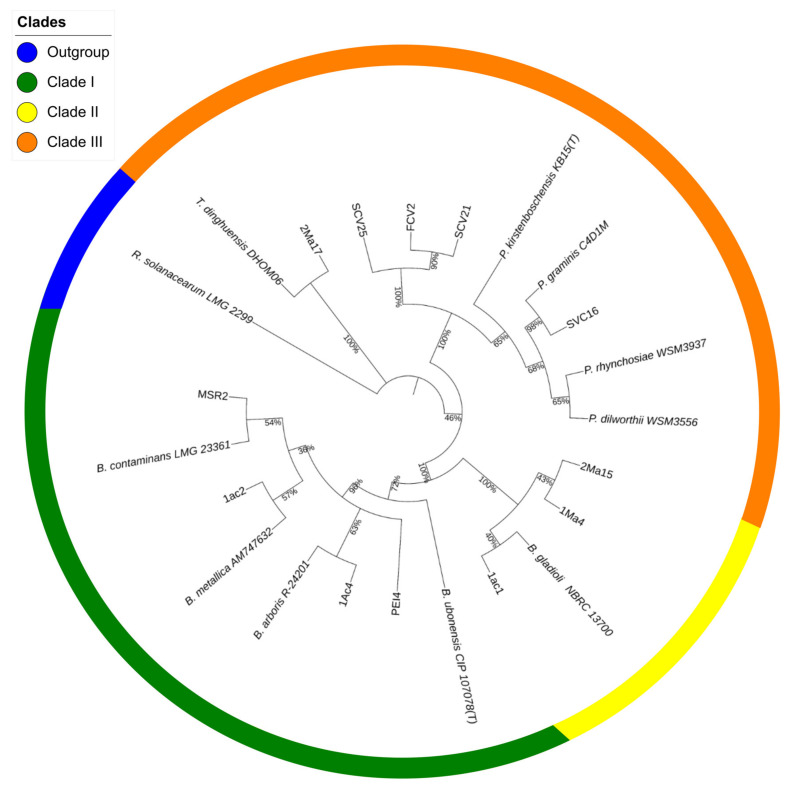
Maximum likelihood phylogenetic tree of rhizosphere *Burkholderia* sensu lato strains. The tree was constructed based on a comparative analysis of 16S rRNA gene sequences. Shown is the phylogenetic position of isolated *Burkholderia* sensu lato strains in relation to *Burkholderia*-type strains. The numbers at the nodes represent percentage levels of bootstrap support from 1000 replications. The sequence of *Ralstonia solanarum* LMG 2299, used as an outgroup, roots the tree.

The sequencing data were used to construct a phylogenetic tree showing the position of the rhizosphere strains in relation to the type strains of species of the *Burkholderia*, *Paraburkholderia*, and *Trinickia* genera that conformed to the tree’s major clusters (Figure 1).

Cluster I showed two subdivisions: one grouped the PEI4 and 1Ac4 strains together with the *B. arboris* R-24201 type strain, while the other placed the 1Ac2 and MSR2 strains along with the *B. contaminans* LMG 23361- and *B. metallica* AM747632-type strains. Cluster II, closely related to the *B. gladioli* NBRC 13700-type strain, was formed with the 1Ma4, 2Ma15 and 1Ac1 strains. Strain 2Ma17 was grouped separately in cluster III with the *T. dinghuensis* DHOM06-type strain. The SCV25, SCV21, FCV2 and SVC16 strains were grouped in a subdivision of this cluster together with the type strains of the *Paraburkholderia* genus. The percentage of similarity of each sequence with respect to the type strains is shown Table S1.

### 3.2. In Vitro Antagonistic Activity against Fusarium spp.

**Figure 2:**
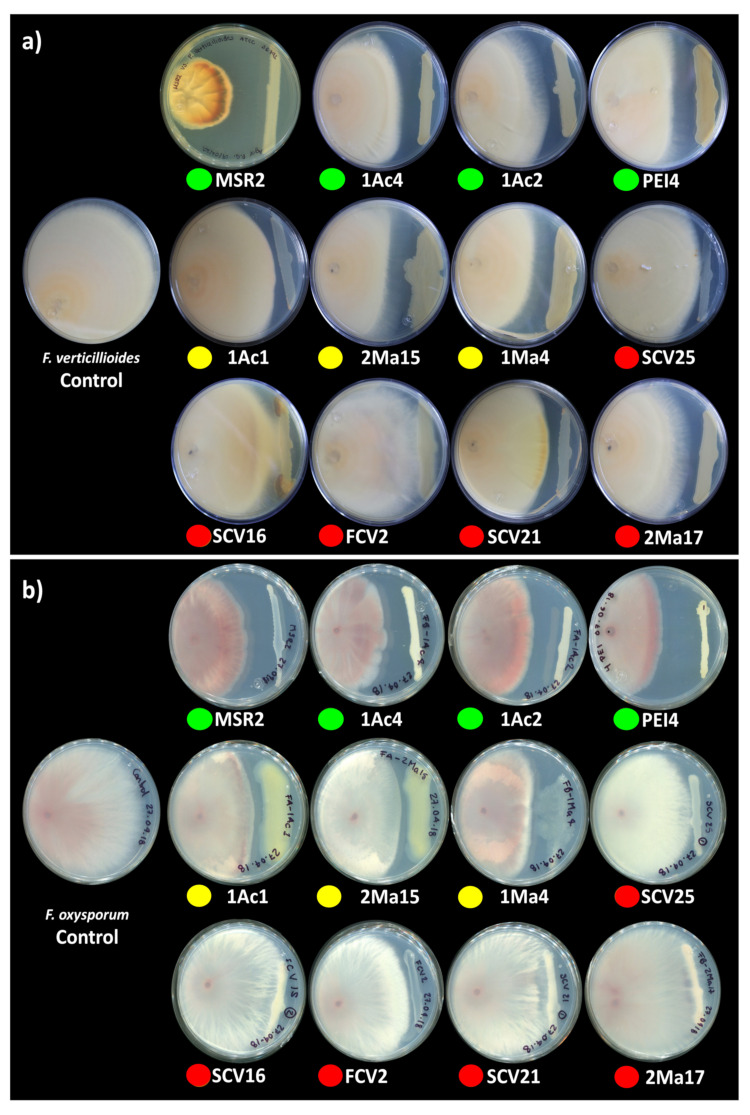
Antagonism of rhizosphere *Burkholderia* sensu lato strains against phytopathogenic *Fusarium*. Antagonism vs. (**a**) *F. verticillioides* MF-257 and (**b**) *F. oxysporum* f. sp. *radicis-lycopersici* was determined by the capacity of rhizosphere *Burkholderia* strains to inhibit their radial hyphal growth when placed in direct confrontation. The single plates in the left end of the image correspond to the negative controls. The colored circles below the plates represent the different *Burkholderia* sensu lato groups: green = Clade I; yellow = Clade II; and orange-red = Clade III. The results shown are representative of experiments that were performed with five (*F. verticillioides*) and three (*F. oxysporum*) replicates per bacterial isolate.

The potential biological control capacity of each *Burkholderia* sensu lato strain was tested by measuring their capability to inhibit the hyphal growth of *F. verticillioides* MF-257 and *F. oxysporum* f. sp. *radicis-lycopersici*. The antagonism of *Burkholderia* sensu lato strains against these phytopathogens was scored by measuring the inhibition zones produced between the fungal mycelium and the bacterial streaks (Figure 2).

**Figure 3:**
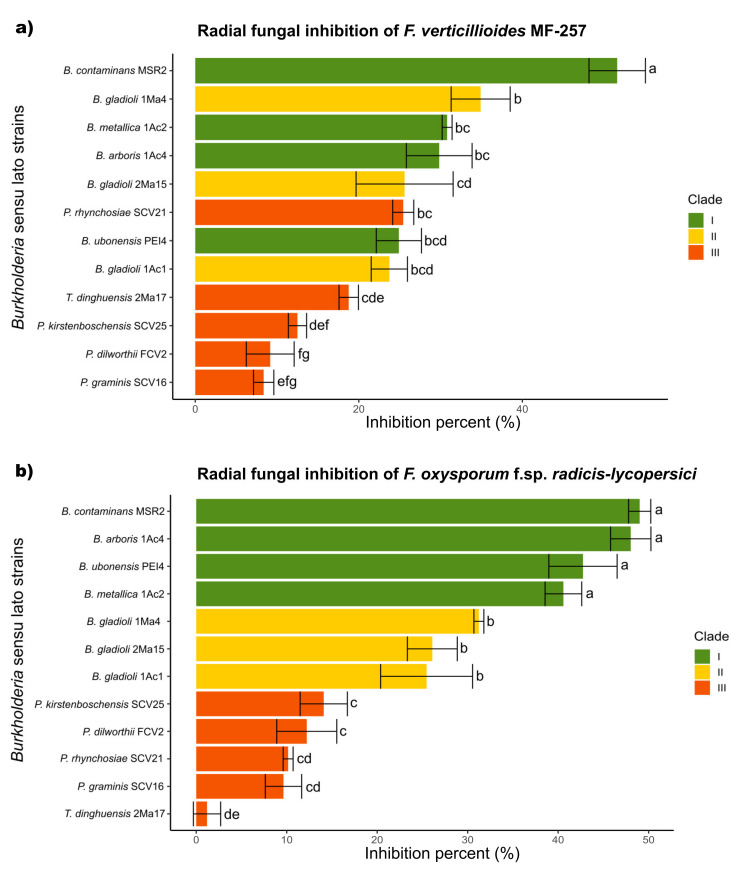
Antifungal activity of *Burkholderia* sensu lato strains against *Fusarium* pathogens. The colored bars represent the percentage of inhibition produced against (**a**) *F. verticillioides* MF-257 and (**b**) *F. oxysporum* f. sp. *radicis*-*lycopersici* by *Burkholderia* sensu lato bacteria isolated from the rhizosphere of maize plants. The bar colors represent their different phylogenetic classifications: green = Clade I; yellow = Clade II; and orange-red = Clade III. Data are the mean values (±std. error) representative of experiments that were performed with five (*F. verticillioides*) and three (*F. oxysporum*) biological replicates per bacterial isolate. Different letters alongside the inhibition percent values represent statistically significant differences (one-way ANOVA, Tukey test, *p* < 0.05).

In general, the mycelial radial growth of both pathogenic fungi was reduced by all the *Burkholderia* sensu lato strains evaluated. The inhibition percent ranged from 3 to 52%, as summarized in Figure 3. *Burkholderia contaminans* MSR2 produced the significantly highest inhibition of the radial growth of *F. verticillioides* (52.0 ± 1.7) and *F. oxysporum* (48.9 ± 1.4%). Furthermore, visible changes in mycelial coloration were observed when *B. contaminans* MSR2 was confronted with each phytopathogen, as shown in Figure 2. The *Burkholderia arboris* 1Ac4, *B. metallica* 1Ac2 and *B. ubonensis* PEI4 strains also had elevated antagonistic activity (i.e., 41–47%) against *F. oxysporum*, while their capacity to suppress the mycelial growth of *F. verticillioides* was lower (i.e., 25–30%). The least inhibitory activity was observed in the clade grouping the *Paraburkholderia* species (Figure 2 and Figure 3).

### 3.3. Plant Growth-Promoting Capability

The in vitro plant growth promotion attributes of the *Burkholderia* sensu lato strains are presented in Table 1. Significant IAA production was detected in all clade II strains. *Burkholderia gladioli* 1Ma4 at 114.6 ± 3 μg/mL was, by far, the highest IAA producer among all the rhizosphere *Burkholderia* sensu lato strains analyzed, whose IAA levels fluctuated between 0.5 and 10.2 µg/mL. Seven *Burkholderia* sensu lato strains were able to solubilize inorganic phosphate. Although phosphate-solubilizing strains were distributed in all *Burkholderia* groups, a significantly major solubilization capability was observed in clade III, specifically in *P. rhynchosiae* SCV2, *P. graminis* SCV16 and *P. kirstenboschensis* SCV25. The ARA yielded nitrogenase activity that ranked between 8.2 and 12.5 nmol/mL·h. The highest activity was detected in the *B. gladioli* 1Ac1 strain, whereas the EPS qualitative screening indicated that eight *Burkholderia* sensu lato strains were positive for the test, as verified by changes in the colonial morphology and in the culture media (Figure S1).

### 3.4. DIESI-MS Metabolic Footprinting

#### 3.4.1. Principal Component Analysis

**Figure 4:**
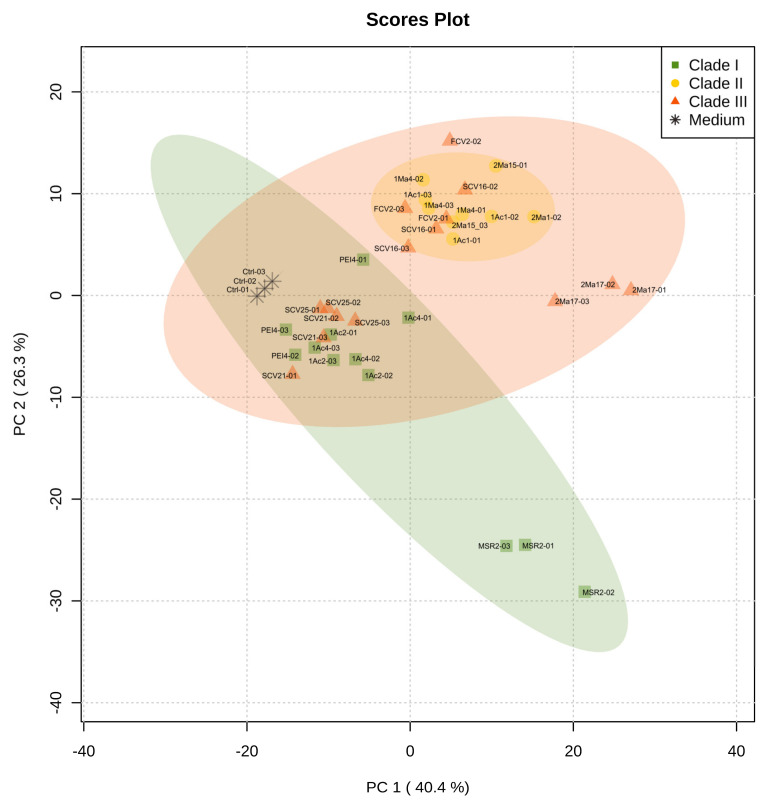
Principal component analysis (PCA) obtained from DIESI-MS analysis of culture extracts of the rhizosphere *Burkholderia* sensu lato strains. Different colored symbols represent the phylogenetic clustering resulting from previous 16S rRNA analysis: green squares (clade I), yellow circles (clade II) and orange-red triangles (clade III). Black asterisks correspond to uninoculated M9 medium controls.

Metabolic footprinting of *Burkholderia* sensu lato strains by DIESI-MS resulted in mass spectra that highlighted the similarities and differences between the 12 strains analyzed (Figure S2). After data binning and filtering, 364 ions within an *m*/*z* range of 200–800 were selected for further analysis. Exo-metabolome comparisons among *Burkholderia* strains were performed by unsupervised multivariate PCA to assess the similarities between the bacteria. As illustrated in Figure 4, the most important sources of data variability (66.7%) were explained by components PC1 (40.4%) and PC2 (26.3%). Clade I and II strains clearly separated into two well-defined groups. In contrast, the bacteria of clade III grouped with the isolates belonging to either clade I or clade II. Thus, *P. kirstenboschensis* SCV25 and *P. rhynchosiae* SCV21 were grouped together with members of the clade I strains, whereas *P. dilworthii* FCV2 and *P. graminis* SCV16 were closer to the strains of clade II. The *T. dinghuensis* 2Ma17 and *B. contaminans* MSR2 strains clearly separated from the rest of the *Burkholderia* strains (Figure 4).

#### 3.4.2. Hierarchical Clustering Analysis

**Figure 5:**
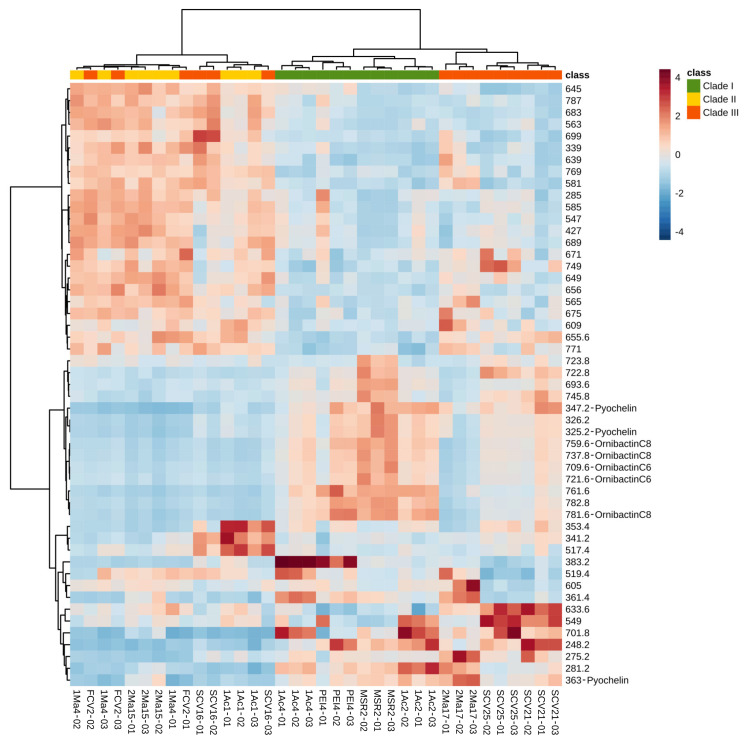
Metabolic heat map based on DIESI-MS data of 50 discriminant ions selected by using the random forest model. The Euclidean distance and Ward’s algorithm were used for the hierarchical classification of rhizosphere *Burkholderia* sensu lato strains, listed on the bottom end of the chart, based on the respective abundance of the discriminant ions selected. Some of these were assigned an identity, as shown for ions corresponding to pyochelin and ornibactin C8 and C6. The colors at the top of the chart are representative of the grouping of the different *Burkholderia* sensu lato strains: clade I (green), clade II (yellow) and clade III (orange-red). The colored bars (in the blue-red range) indicate the abundance of each metabolite ion, where red indicates higher signal intensity.

To categorize and select the important metabolic features of the *Burkholderia* sensu lato strains, 50 discriminant ions were chosen according to their mean decrease accuracy valued, as determined by the RF model. This strategy’s objective was to reduce the data complexity and detect significant differences among the DIESI-MS profiles. The Euclidian distance and Ward’s algorithm were applied to the ions selected in order to obtain the chemical profiles of the *Burkholderia* sensu lato strains. The results are presented as a heatmap depicting the abundance of these metabolite ions in the strain’s cultures (Figure 5). Hierarchical clustering of the *Burkholderia* sensu lato strains (top of Figure 5) showed that all clade I strains (*B. contaminans* MSR2, *B. arboris* 1Ac4, *B. metallica* 1Ac2 and *B. ubonensis* PEI4) grouped together. Conversely, some clade II strains (e.g., *B. gladioli* 1Ac1, *B. gladioli* 2Ma15 and *B. gladioli* 1Ma4) clustered together with two clade III strains (*P. dilworthii* FCV2 and *P. graminis* SCV16), while the rest of the clade III strains (i.e., *P. rhynchosiae* SCV21, *P. kirstenboschensis* SCV25 and *T. dinghuensis* 2Ma17) formed a distinct and chemically related group. As observed, the grouping of these *Burkholderia* sensu lato strains based on metabolic attributes showed variations from the phylogenetic classification based on the analysis of their respective 16S rRNA sequences. This difference most likely reflected the metabolic diversity that characterizes the *Trinickia*, *Burkholderia* and *Paraburkholderia* genera [53].

#### 3.4.3. DIESI-MSQD Analysis and MS/MS Siderophore Identification

Representative DIESI-MS spectra of the 200–800 *m*/*z* region showed the most intense ions corresponding to the exo-metabolites secreted to the culture medium by each strain (Figure S2). The identity of the most intense ions, as determined by the RF model (Figure 5), was obtained by their subsequent fragmentation (see Table 2 and Table S2 [54,55,56]). Remarkably, *B. contaminans* MSR2 generated ion signals with *m*/*z* values of 737.7, 759.7 and 781.6, whose maximal intensity was confirmed by an ANOVA test. Interestingly, the first ion was found to correspond to the [M+H]^+^ adduct of the ornibactin C8 siderophore, while the 759 and 781 *m*/*z* signals corresponded to the sodium (+23 Da) and potassium (+39 Da) adducts of ornibactin C8, respectively (Figure 5 and Table 2).

**Figure 6:**
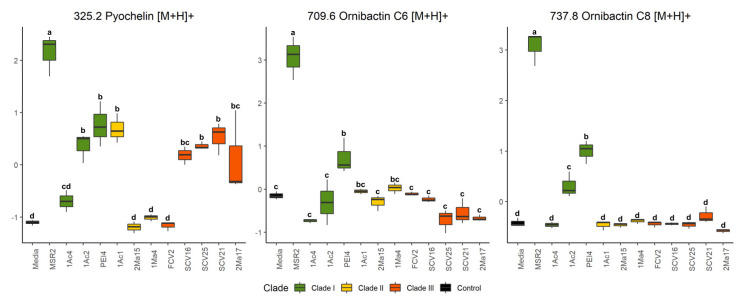
Quantification of siderophore accumulation in the culture media of rhizosphere *Burkholderia* sensu lato strains. The box plots represent the normalized intensities of the ion signals identified as part of the pyochelin (*m*/*z* 325.2), ornibactin C6 (709.6) and ornibactin C8 (737.8) siderophores. These were detected in the culture media used to grow different rhizosphere *Burkholderia* sensu lato strains. Different letters over the box plots represent statistically significant differences (one-way ANOVA, Tukey test, *p* < 0.05). The box plot colors are representative of the grouping of the different *Burkholderia* sensu lato strains: clade I (green), clade II (yellow) and clade III (orange-red).

In addition to *B. contaminans* MSR2, the signature ions of this metabolite were also intense in clade I *B. ubonensis* PEI4 and *B. arboris* 1Ac2 (Figure 6). The secretion of ornibactin C6 (709 *m*/*z*, [M+H]^+^ and 721 *m*/*z*, [M+Na]^+^; Table 2) was also detected in the cultures of the *B. contaminans* MSR2 strain, while no significant accumulation of these siderophore signals was detected in the rest of clade I strains (Figure 6). The intensity of these ions was also very weak in the samples of strains conforming clades II and III. Conversely, the pyochelin siderophore (i.e., 325 [M+H]^+^ and 347 [M+Na]^+^ *m*/*z* ions; Table 2) was produced by most of the strains analyzed.

The ANOVA test showed that, like ornibactin C6 and C8, the MSR2 strain secreted the highest concentration of pyochelin compared with all other *Burkholderia* strains analyzed (Figure 6). Among these, the clade III *Paraburkholderia* strains secreted intermediate amounts of the latter siderophore. Included in this group were *P. graminis* SCV16, *P. rhynchosiae* SCV21, *P. kirstenboschensis* SCV25 and *T. dinghuensis* 2Ma17 but not *P. dilworthii* FCV2. A characteristic of clade II strains was their inability to secrete at least one of these three siderophores.

## 4. Discussion

This study identified rhizobacteria associated with suppressive soils able to reduce the incidence of highly damaging fungal infections of cultivated maize in central Mexico. It concentrated on those caused by *F. verticillioides* and *F. oxysporum*, which are known to severely impact worldwide maize production in tropical and subtropical latitudes [3,57]. *Fusarium verticillioides* is frequently reported as the causative agent of severe maize plant diseases characterized by extensive stalk, ear or root decay that may also lead to reduced grain quality [58,59]. Consistent with the high diversity of *Burkholderia* bacteria that is found in soils [60], the analysis of several maize rhizospheres obtained from plants sampled from diverse agricultural soils of the Bajío region of central Mexico led to the isolation and identification of several *Burkholderia* sensu lato strains. Traditional sequence analysis of the 16S rRNA gene was used for the identification of these strains, which were further subdivided into three clades on the basis of their sequence similarity with the strain types. This classification grouped strain 2Ma17 and the *T. dinghuensis* DHOM06-type strain within clade III, together with the *Paraburkholderia* strains. However, the deep branching observed suggests that the alternative of placing the 2Ma17 strain in a different clade is a plausible possibility. This proposal is supported by a recent study in which a maximum likelihood analysis of conserved genes from more than 100 *Burkholderia* sensu lato species strongly supported the grouping of the novel *Mycetohabitans* and *Trinickia* genera described into two distinct and unique clades [53]. Similar to the present study, the *Trinickia* cluster was positioned between those grouping the *Burkholderia* and *Paraburholderia* strains.

This and other molecular biology methods have also been used to identify *Burkholderia* bacteria [61,62]. However, they appear to be insufficient for correctly classifying those belonging to the Bcc, whose taxonomic status remains problematic [63]. In this respect, the analysis of whole genome sequences was proposed as the method of choice for the identification of Bcc bacteria and others that are difficult to identify by conventional techniques [64]. On the other hand, other more practical approaches based on the use of mass spectrometry methods have been developed to obtain rapid and reliable diagnostic results in clinical microbiology. These are based on the generation and interpretation of mass spectra for the fast identification of indicative pathogenic microorganisms, including bacteria relevant to this study, such as *B. contaminans* [65,66,67,68,69,70]. In this context, the results obtained in the present study revealed that, in contrast to the MALDI-MS-based methods mentioned above, the utilization of untargeted DIESI-MS analysis offered a rapid, accurate and cost-effective alternative for the detection and quantification of siderophores in *Burkholderia* cell-free cultures. Thus, MALDI-TOF analysis of the acidified ethyl acetate extracts of *B. contaminans* MSR2 cell-free extracts incubated in minimum M9 media supplemented with 100 µM of FeCl_3_ to inhibit siderophore synthesis was unable to appropriately identify ornibactin C6, ornibactin C8 and pyochelin in the relatively complex bacterial extracts, even in the absence of Fe^3+^. Moreover, DIESI-MS proved itself to be equivalent to the laborious and time-consuming UPLC-MS analysis for the identification of these siderophores in cell-free bacterial cultures (data not shown).

In accordance with the previously published data, the maize rhizosphere *Burkholderia* strains had properties known to be associated with plant growth promotion [26,71,72,73,74,75]. These were unevenly distributed among the different groups, as shown by (1) the detection of clade II *B. gladioli* 1Ma4 as a lone IAA hyper-accumulator; (2) the dispersal of PS and nitrogen-fixing strains in clades I and III and (3) the dominance of EPS production in the clade I strains. The detection of IAA and N-fixing activity in Bcc strains belonging to clade I and in the two others was in accordance with the studies by Suárez-Moreno et al. [26], Caballero-Mellado et al. [72], Angus et al. [73], Martínez-Aguilar et al. [76] and Estrada de los Santos et al. [77], while PS and EPS production by Bcc and *Paraburkholderia* bacteria similar to those grouped in clades I and III, respectively, was associated with the growth promotion of C4 plants [77,78,79,80,81]. These results strongly suggest that these *Burkholderia* strains could be important maize plant growth-promoting bacteria. Some of them also revealed a potential capacity to protect these plants against aggressive soil-borne fungi, such as specific *Fusarium* pathogens.

Thus, the in vitro confrontation assays indicated that some isolated maize rhizosphere *Burkholderia* strains, mainly *B. contaminans* and *B. arboris*, strongly inhibited the growth of the two pathogenic *Fusarium* genotypes tested. The suppression effect was associated with the accumulation of ornibactin and pyochelin siderophores, in accordance with reports linking the antifungal behavior of some *Burkholderia* strains via iron sequestration through the synthesis of ornibactin [82,83], malleobactin and pyochelin [19,84]. Supporting evidence for this proposal was provided by a recent study that attributed the antimicrobial activity versus antibiotic resistant pathogens detected in *Burkholderia* sensu stricto bacteria to several metabolites, including the ornibactin and pyochelin siderophores [85]. Additional evidence showing that these metabolites were rarely present in strains classified within the *Paraburkholderia, Trinickia* and other genera was also in agreement with their lack of biocontrol capacity against phytopathogenic fungi, as reported in this and other studies. In this respect, the finding that the pyochelin-producing *Paraburkholderia* and *Trinickia* strains had the lowest inhibition activity versus *Fusarium* suggests that this metabolite, despite its siderophore activity, has a low biocontrol potential, at least against these fungal pathogens. In addition, data from recent reports support the argument that other unidentified metabolites could have been contributing factors to the different antifungal effect observed in the present study between *B. contaminans* and *B. arboris* on one hand and the *Paraburkholderia and Trinickia isolates* on the other. In this respect, the superior *Rhizoctonia*-suppressive activity produced by *P. graminis* PHS1 compared with several other isolates of the *Paraburkholderia* and *Burkholderia* species was found to not depend on diffusible antimicrobial compounds but on the emission of sulfurous volatile compounds [86]. This study also suggested that differences found in the gene clusters encoding polyketide synthases and non-ribosomal peptide synthetases of unknown functions between these bacteria could lead to the synthesis of still unknown metabolites able to increase the suppressive potential of soils against fungal pathogens.

The results of the present study complement related studies that identified certain traits in *Burkholderia* and other rhizosphere bacteria that were linked to the control of *Fusarium*-related infections in maize and other cereals. Thus, activity against *Aspergillus flavus* and *F. verticillioides* was associated to the production of extracellular chitinolytic enzymes or the accumulation of antifungal metabolites, mainly by *Burkholderia* and *Pseudomonas* bacteria [87]. Siderophores and auxins were also identified as components responsible for reducing the disease severity caused by *F. verticillioides* in maize plants [57]. Likewise, two growth-promoting *Burkholderia* strains isolated from maize rhizospheres and identified as *B. cenocepacia and B. contaminans* showed broad spectrum antifungal activity against several pathogenic *Fusarium* species [88], whereas the capacity to degrade fusaric acid by a *B. ambifaria* strain isolated from the rhizosphere of barley contributed to the biocontrol of Fusarium wilt in barley seedlings [75]. Finally, the moderate capacity to inhibit *F. verticillioides* and the plant growth-promoting potential of the *Paraburkholderia* strains reported in this study, in addition to previously reported data [86], indicate that this marginally studied genus represents an interesting subject for future research that could employ untargeted metabolomic approaches as experimental tools. This effort could compensate for the scarce information regarding the metabolites synthesized by the numerous species that conform to the *Paraburkholderia* genus.

From a technical perspective, it may be concluded that the DIESI MS methodology offers a rapid and cost-effective approach for the identification of key metabolite-associated pathogen inhibitory effects and, potentially, the growth promotion capability of novel isolates, such as the rhizosphere *Burkholderia* sensu lato strains described in this study. This was demonstrated by the identification of biocontrol-related metabolites, such as siderophores, which were associated with the capacity of *B. contaminans*, *B. ubonensis* and *B. metallica* to suppress the growth of *Fusarium* phytopathogens.

## Figures and Tables

**Table 1:** Plant growth-promoting activities of rhizosphere *Burkholderia* sensu lato strains.

Strain	IAA (μg/mL)	PS (mg/L)	ARA (nmol/mL·h)	EPs (Qualitative)
*B. contaminans* MSR2	0.5 ± 0.1 ^c^	122 ± 7 ^d^	0 ± 0 ^b^*	+
*B. arboris* 1Ac4	2.3 ± 0.1 ^bc^	64 ± 3 ^e^	0 ± 0 ^b^	+
*B. metallica* 1Ac2	0.6 ± 0.03 ^c^	0 ± 0 ^f^	8.4 ± 0.2 ^a^	+
*B. ubonensis* PEI4	1.3 ± 0.1 ^bc^	0 ± 0 ^f^	8.7 ± 0.06 ^a^	+
*B. gladioli* 1Ac1	0 ± 0 ^c^	171 ± 12 ^c^	12.5 ± 1.2 ^a^	−
*B. gladioli* 2Ma15	0.93 ± 0.2 ^c^	5.1 ± 0.2 ^f^	8.6 ± 0.14 ^a^	+
*B. gladioli* 1Ma4	114.6 ± 3 ^a^	37 ± 2 ^ef^	8.2 ± 0.1 ^a^	+
*P. graminis* SCV16	10.2 ± 0.7 ^b^	409 ± 2 ^ab^	0 ± 0 ^b^	−
*P. dilworthii* FCV2	1.4 ± 0.1 ^bc^	29 ± 2 ^ef^	0 ± 0 ^b^	+
*P. kirstenboschensis* SCV25	1.6 ± 0.3 ^bc^	391 ± 5 ^b^	0 ± 0 ^b^	−
*P. rhynchosiae* SCV21	1.2 ± 0.04 ^c^	441 ± 12 ^a^	8.8 ± 0.1 ^a^	−
*T. dinghuensis* 2Ma17	1.1 ± 0.2 ^c^	3.3 ± 0.3 ^f^	8.3 ± 0.04 ^a^	+

IAA = indoleacetic acid; PS = phosphate solubilization; ARA = acetylene reduction assay; and EPS = exopolysaccharides production (based on culture media color change), where − = negative and + = positive tests, respectively. Data are the mean values of three biological replicates. * Different letters alongside the IAA, PS and ARA values represent statistically significant differences (one-way ANOVA, Tukey test, *p* < 0.05).

**Table 2:** Metabolites secreted by *Burkholderia* sensu lato maize rhizosphere strains as identified by MS/MS analysis.

*m*/*z*	Ion	Molecular Weight	Identity	Molecular Formula
190	[M-135+H]^+^	324.413	Pyochelin	C_14_H_16_N_2_O_3_S_3_
325.2	[M+H]^+^			
347	[M+Na]^+^			
363	[M+K]^+^			
709	[M+H]^+^	708.767	Ornibactin C6	C_28_H_52_N_8_O_13_
721	[M+Na]^+^			
737	[M+H]^+^	736.821	Ornibactin C8	C_30_H_56_N_8_O_13_
759	[M+Na (23)]^+^			
760	[M+Na (23)]^+^			
775	[M+K (39)]^+^			

## Data Availability

The bacterial 16S rRNA gene sequencing data reported in this paper have been deposited in the GenBank^®^ genetic sequence database under the accession numbers MZ275276 to MZ275286.

## Supplementary Materials

The following are available online at https://www.mdpi.com/article/10.3390/microorganisms9102061/s1. Figure S1: Congo red agar assay for the qualitative detection of exopolysaccharide (EPS) production. Figure S2: DIESI-MSQD spectra of exometabolomes from rhizosphere *Burkholderia* sensu lato strains. Table S1: Phylogenetic similarity of bacteria isolated from maize rhizospheric soil. Table S2: Fragmentation ion pattern of selected signals obtained from DIESI-MS analysis of rhizospheric *Burkholderia* sensu lato culture supernatants.
