# Supplementary material for: Metabolic Footprints of Burkholderia Sensu Lato Rhizosphere Bacteria Active against Maize Fusarium Pathogens
Source: Microorganisms. 2021 Sep 29;9(10):2061. doi: 10.3390/microorganisms9102061 (PMC8538949; doi:10.3390/microorganisms9102061)
Supplement: Supplementary file 1 [file microorganisms-09-02061-s001.zip › microorganisms-1359620-supplementary.pdf]

Supplementary Materials

Metabolic foot-prints of *Burkholderia* sensu lato rhizosphere bacteria active against maize *Fusarium* pathogens.

Guadalupe C. Barrera-Galicia [1], Héctor A. Peniche-Pavía [1], Juan J. Peña-Cabriales [1], Sergio A. Covarrubias [2], José A. Vera-Núñez [2] and John P. Délano-Frier [1,*]

[1] Centro de Investigación y de Estudios Avanzados del Instituto Politécnico Nacional, Unidad Irapuato, Libramiento Norte Carretera Irapuato León Kilómetro 9.6, Carretera Irapuato León, Irapuato 36824, Guanajuato, México

[2] Universidad Autónoma de Zacatecas, Área de Ciencias de la Salud. Carretera Zacatecas-Guadalajara Km. 6, ejido "La Escondida". Ciudad Universitaria campus Siglo XXI, Zacatecas 98160, Zacatecas, México

*Correspondence: john.delano@cinvestav.mx; Tel.: +52 462-623-9600

This Supporting Information including:

Figure S1. Congo red agar assay for the qualitative detection of exo-polysaccharides (EPS) production.

Figure S2. DIESI-MSQD spectra of exo-metabolomes from rhizosphere *Burkholderia* sensu lato strains.

Table S1. Phylogenetic similarity of bacteria isolated from maize rhizospheric soil.

Table S2. Fragmentation ion pattern of selected signals obtained from DIESI-MS analysis of rhizospheric *Burkholderia* sensu lato culture supernatant.

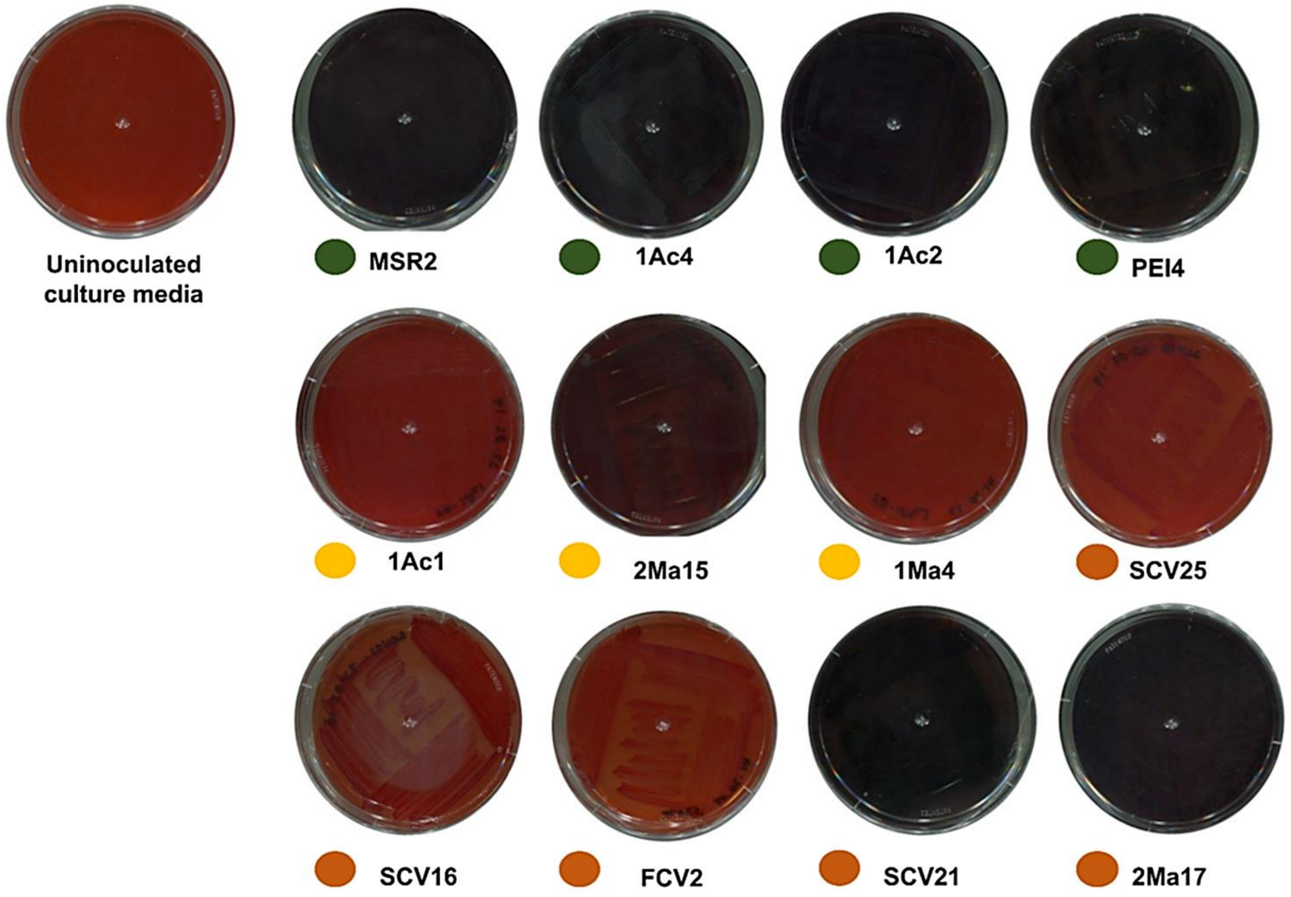

Figure S1. Congo red agar assay for the qualitative detection of exo-polysaccharides (EPS) production. An EPS positive result was indicated by the development of black colonies (in dark Petri dishes), while the colonies of non-slime producers remained pink (in red Petri dishes). The single plate in the upper-left side of the image corresponds to the negative control (i.e., uninoculated media). The colored circles below the plates represent the different *Burkholderia* sensu lato groups: green = Clade I; yellow = Clade II, and orange = Clade III.

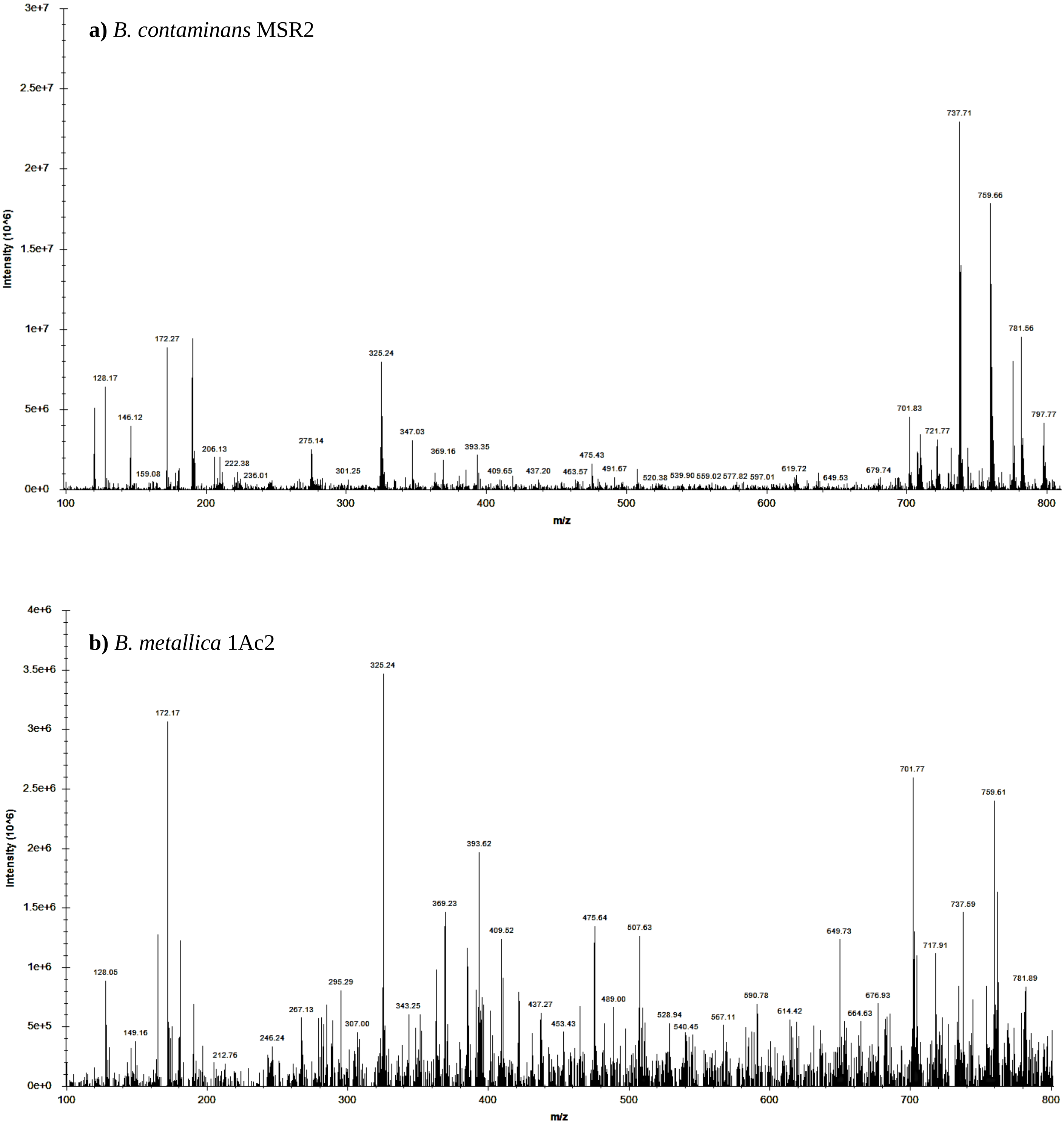

a) B. contaminans MSR2
Intensity (10^6)
m/z
737.71
759.66
781.56
797.77
701.83
721.77
679.74
649.53
619.72
597.01
577.82
559.02
539.90
520.38
491.67
475.43
463.57
437.20
409.65
393.35
369.16
347.03
325.24
301.25
275.14
236.01
222.38
206.13
172.27
159.08
146.12
128.17
b) B. metallica 1Ac2
325.24
172.17
701.77
759.61
393.62
369.23
737.59
475.64
507.63
409.52
649.73
717.91
128.05
781.89
295.29
590.78
489.00
676.93
437.27
267.13
343.25
614.42
664.63
307.00
528.94
567.11
453.43
540.45
149.16
246.24
212.76

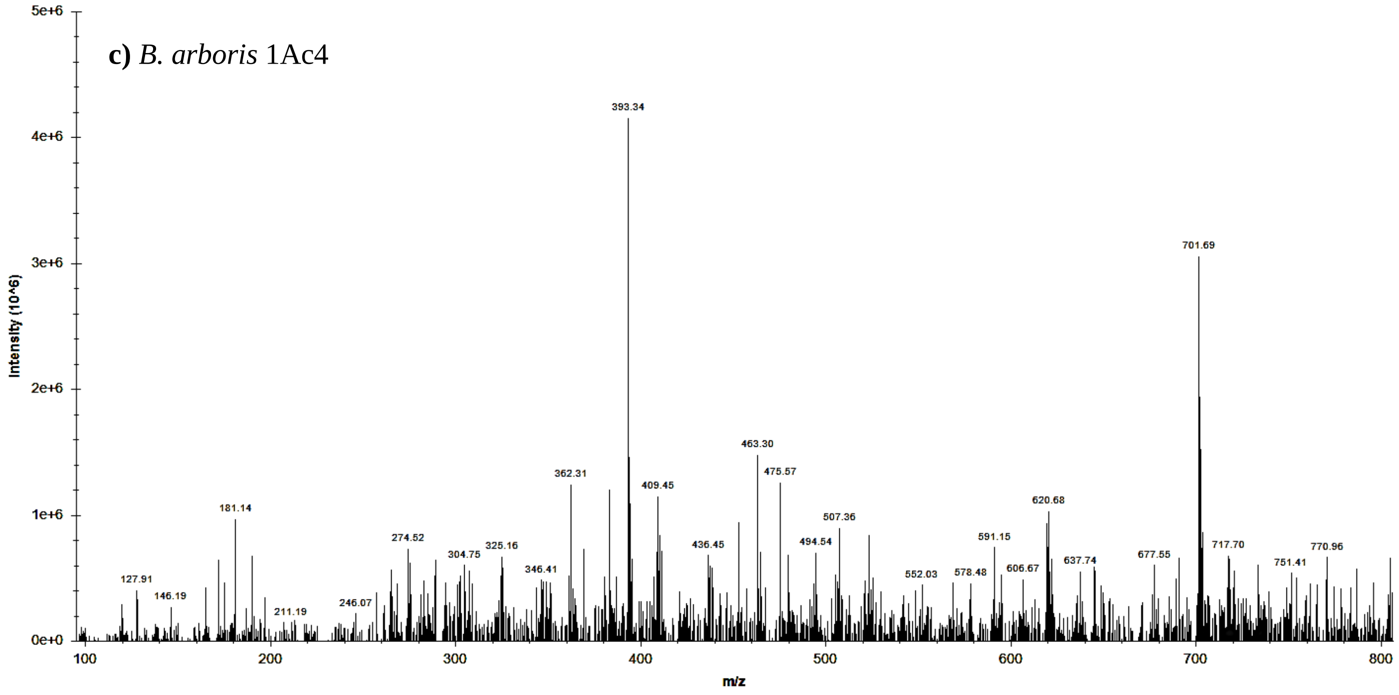
c) B. arboris 1Ac4
Intensity (10^6)
m/z
393.34
701.69
463.30
475.57
362.31
409.45
181.14
620.68
507.36
274.52
591.15
717.70
770.96
436.45
494.54
325.16
304.75
637.74
677.55
751.41
346.41
606.67
578.48
552.03
127.91
146.19
246.07
211.19

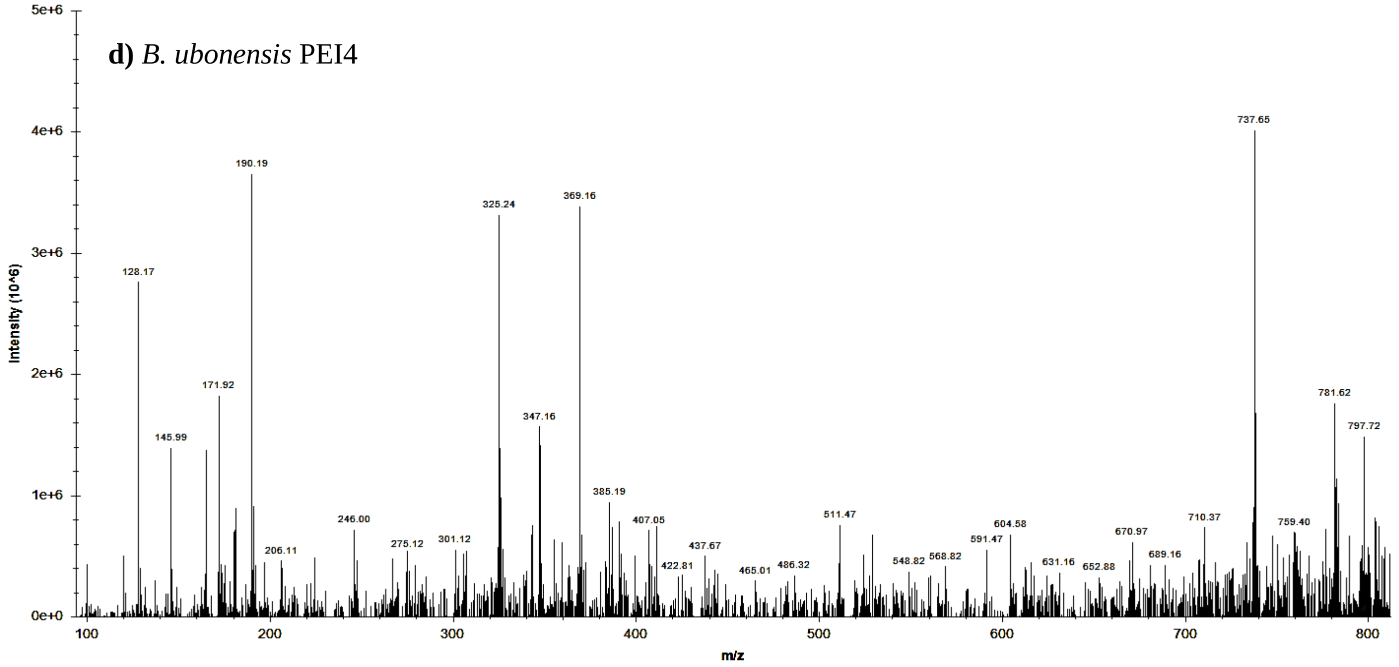
d) B. ubonensis PEI4
Intensity (10^6)
m/z
737.65
190.19
369.16
325.24
128.17
171.92
781.62
797.72
145.99
347.16
385.19
511.47
246.00
407.05
710.37
759.40
604.58
670.97
301.12
275.12
206.11
437.67
591.47
689.16
548.82
568.82
631.16
652.88
422.81
486.32
465.01

e) *B. gladioli* 1Ac1

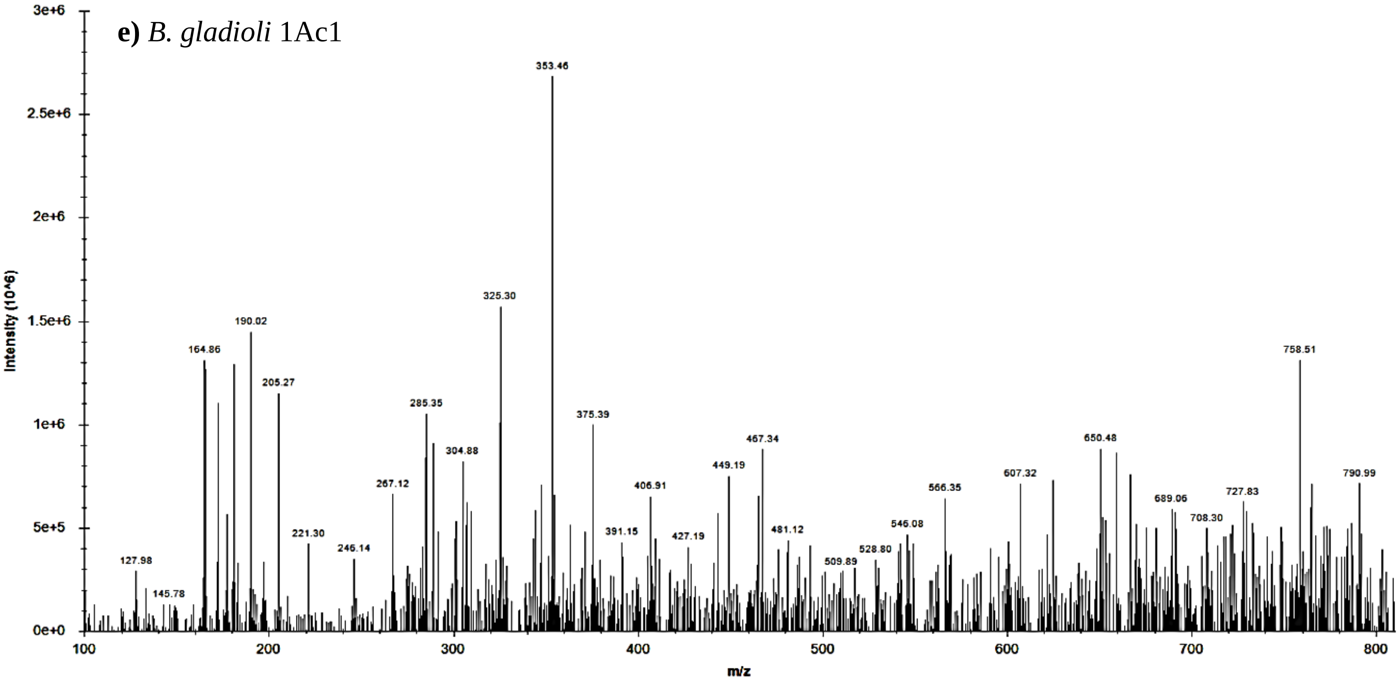

f) *B. gladioli* 2Ma15

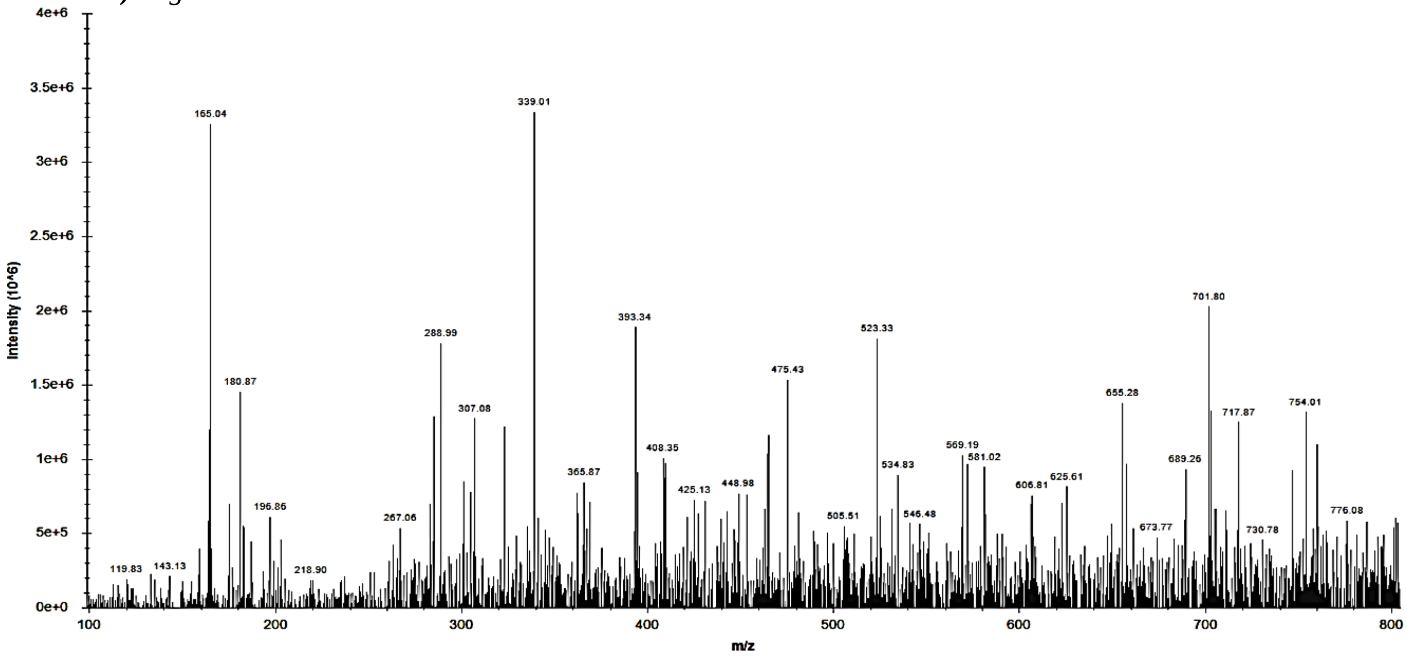

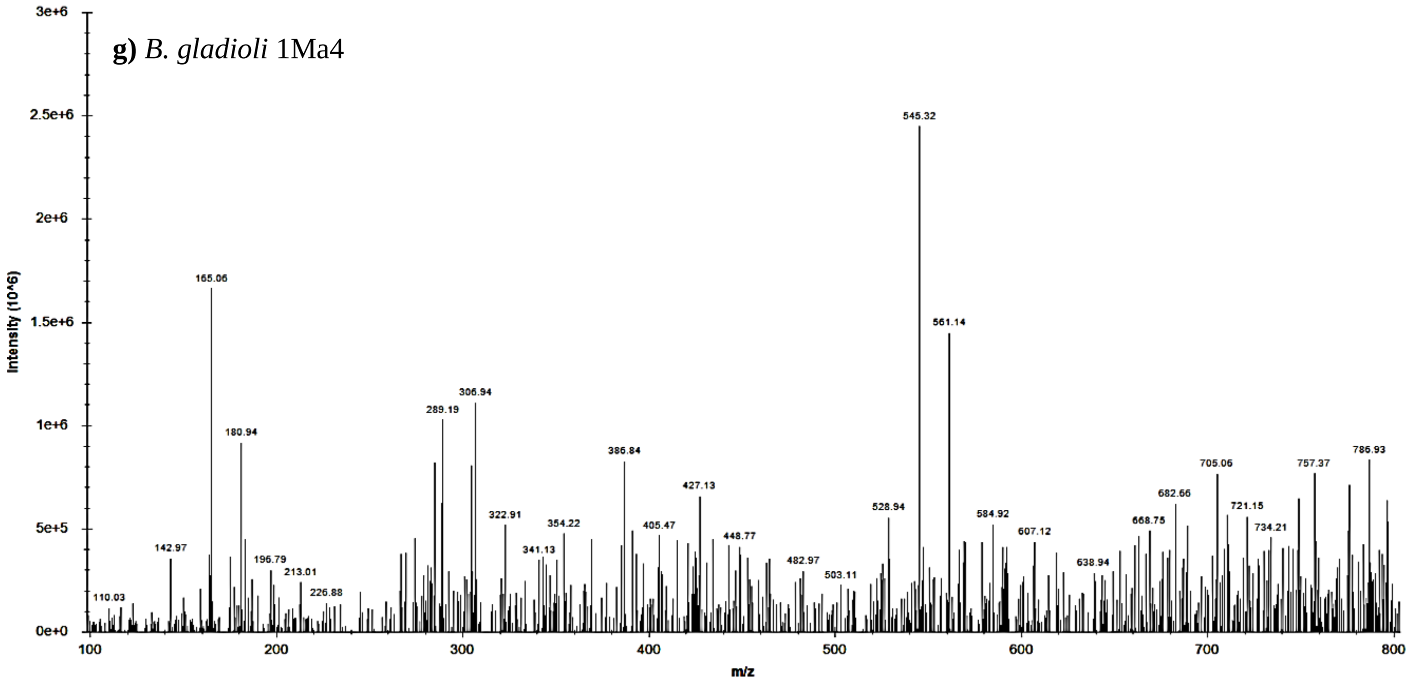
g) B. gladioli 1Ma4
Intensity (10^6)
3e+6
2.5e+6
2e+6
1.5e+6
1e+6
5e+5
0e+0
m/z
100
200
300
400
500
600
700
800
545.32
561.14
165.06
180.94
142.97
110.03
196.79
213.01
226.88
289.19
306.94
322.91
341.13
354.22
386.84
405.47
427.13
448.77
482.97
503.11
528.94
584.92
607.12
638.94
668.75
682.66
705.06
721.15
734.21
757.37
786.93

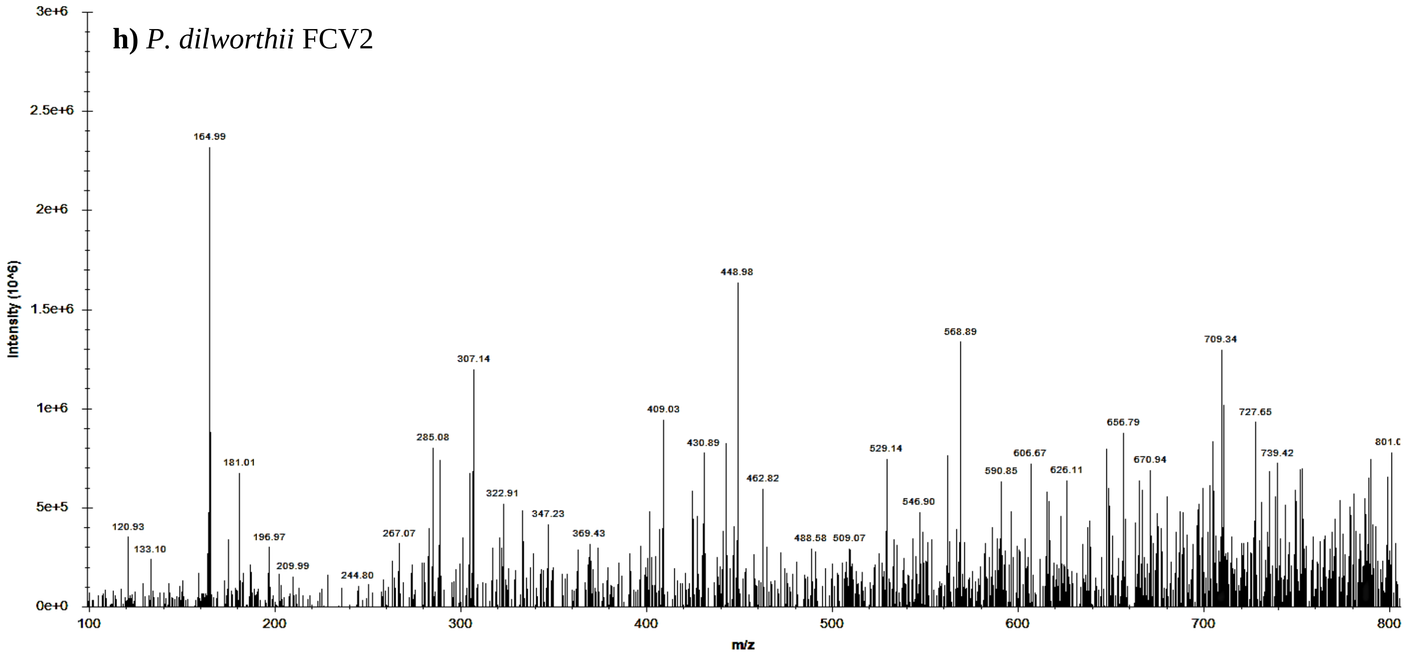
h) P. dilworthii FCV2
Intensity (10^6)
3e+6
2.5e+6
2e+6
1.5e+6
1e+6
5e+5
0e+0
m/z
100
200
300
400
500
600
700
800
164.99
181.01
120.93
133.10
196.97
209.99
244.80
267.07
285.08
307.14
322.91
347.23
369.43
409.03
430.89
448.98
462.82
488.58
509.07
529.14
546.90
568.89
590.85
606.67
626.11
656.79
670.94
709.34
727.65
739.42

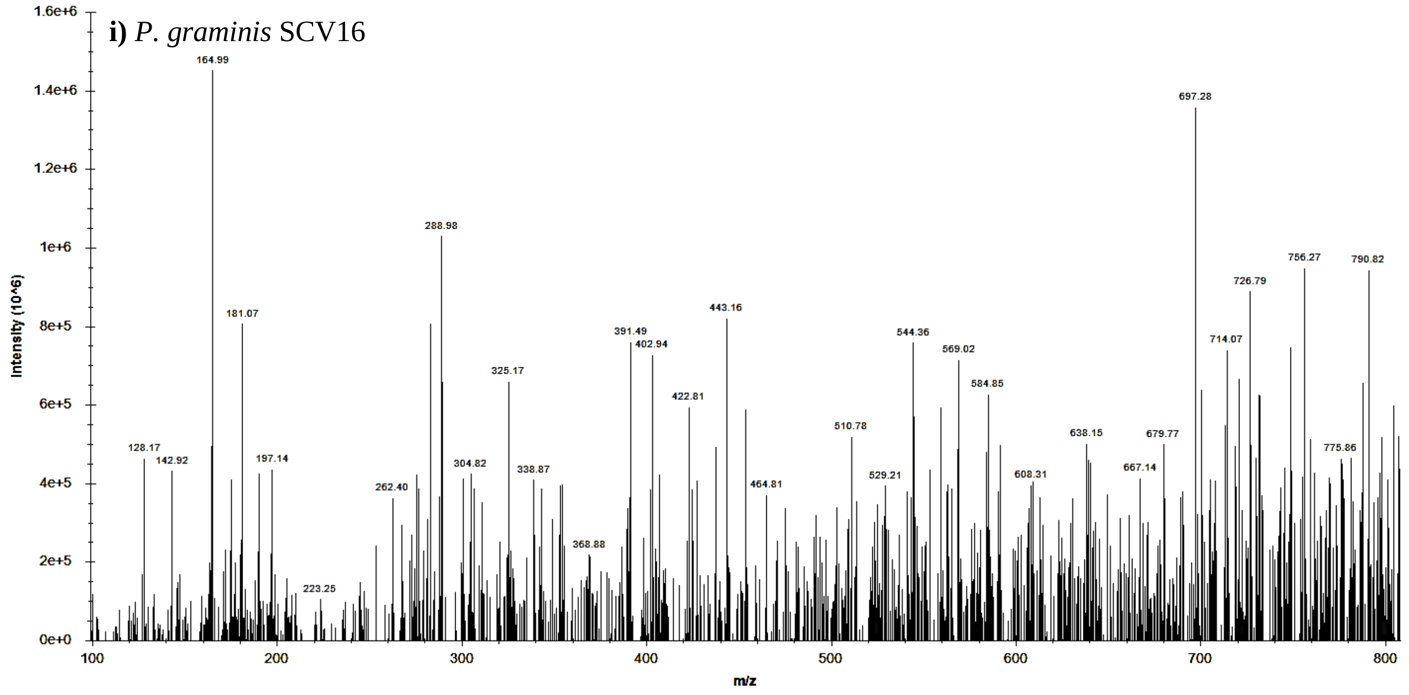
i) P. graminis SCV16
1.6e+6
1.4e+6
1.2e+6
1e+6
8e+5
6e+5
4e+5
2e+5
0e+0
Intensity (10^6)
164.99
697.28
288.98
756.27
790.82
726.79
181.07
443.16
714.07
391.49
544.36
402.94
569.02
325.17
584.85
422.81
510.78
638.15
679.77
128.17
142.92
197.14
775.86
304.82
338.87
529.21
608.31
667.14
262.40
464.81
368.88
223.25
100
200
300
400
500
600
700
800
m/z

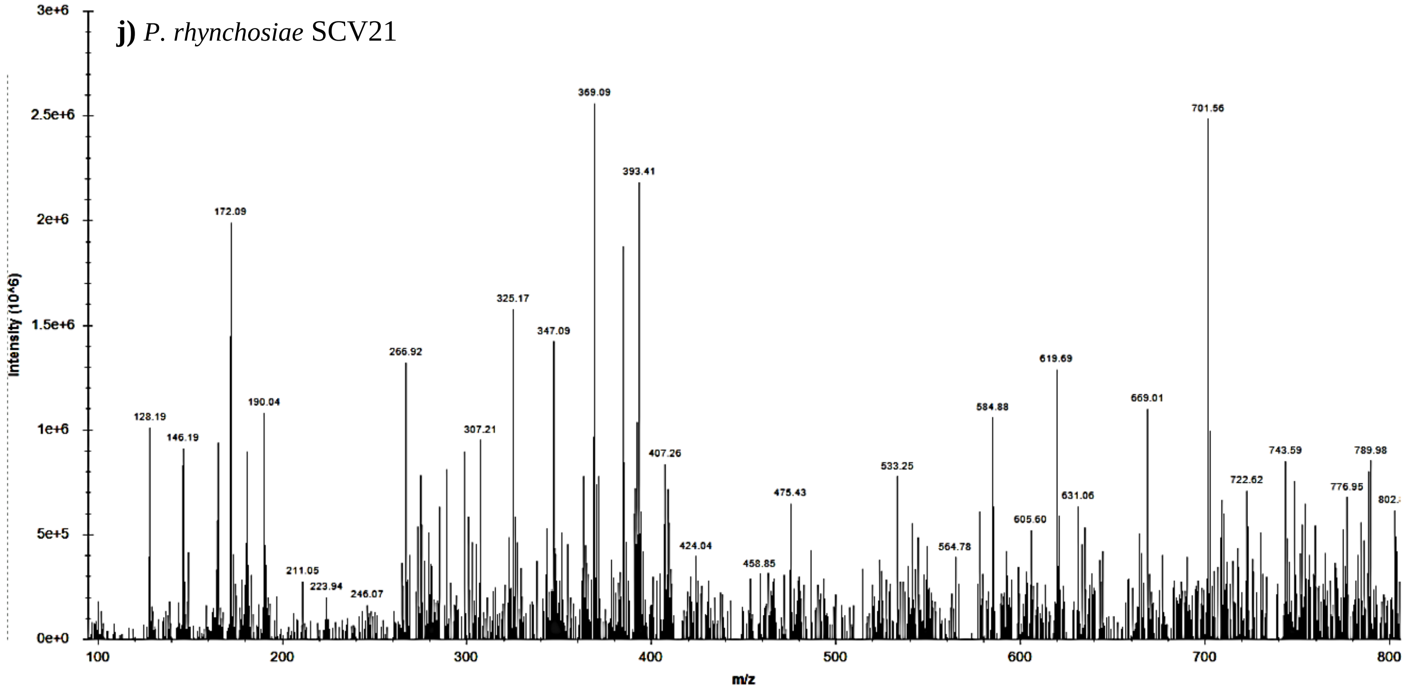
j) P. rhynchosiae SCV21
3e+6
2.5e+6
2e+6
1.5e+6
1e+6
5e+5
0e+0
Intensity (10^6)
369.09
701.56
393.41
172.09
325.17
347.09
266.92
619.69
190.04
584.88
669.01
128.19
307.21
146.19
407.26
743.59
789.98
533.25
722.62
475.43
776.95
631.06
802.
605.60
424.04
564.78
458.85
211.05
223.94
246.07
100
200
300
400
500
600
700
800
m/z

k) *P. kirstenboschensis* SCV25

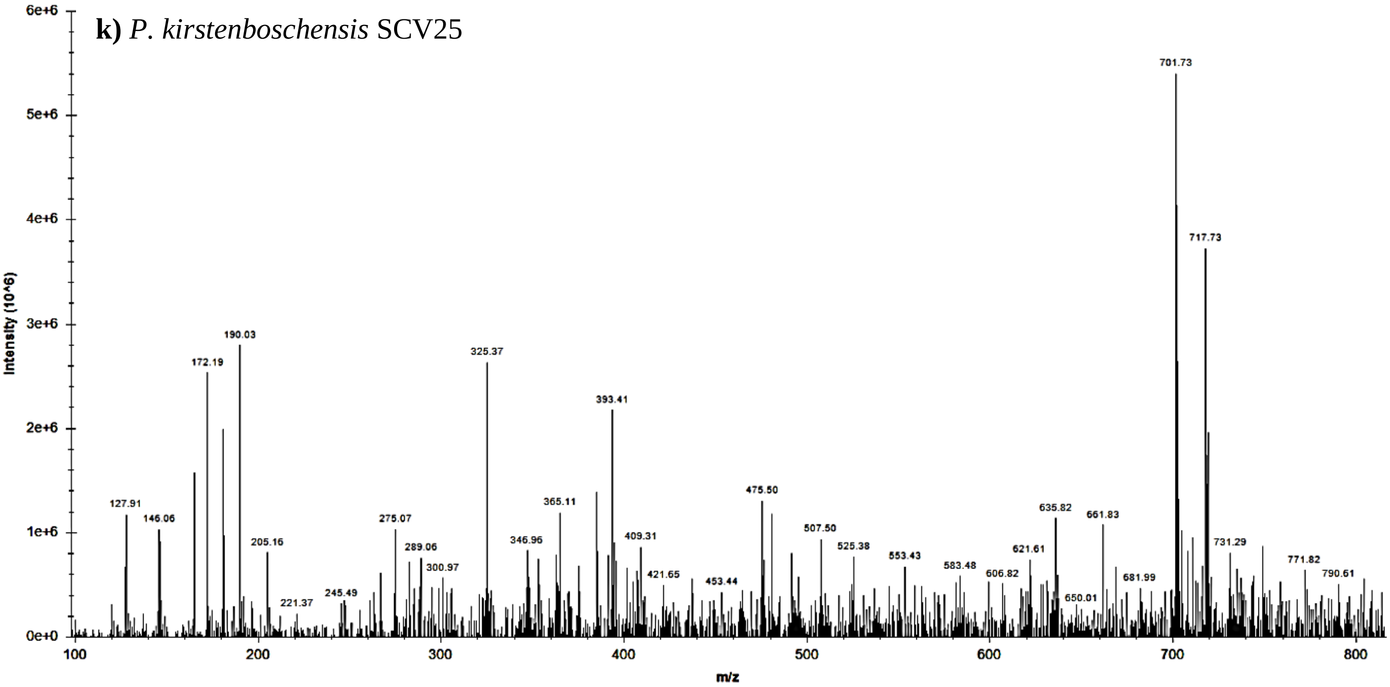

l) *T. dinghuensis* 2Ma17

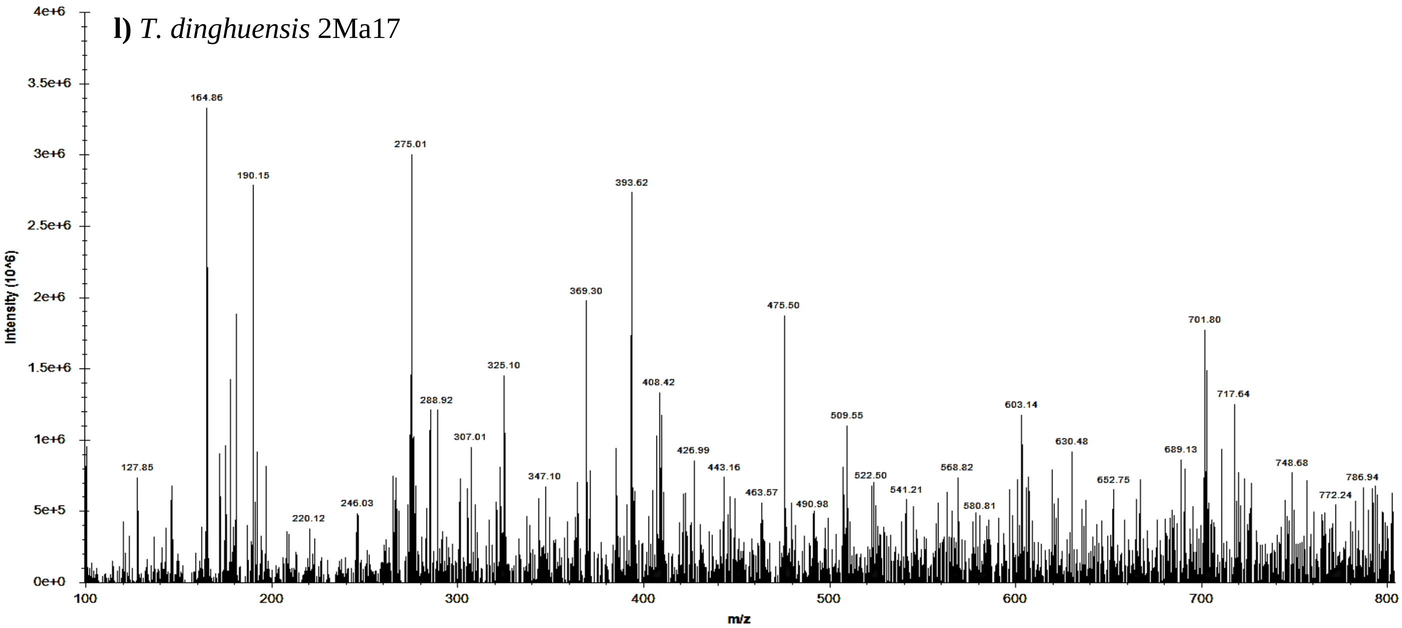

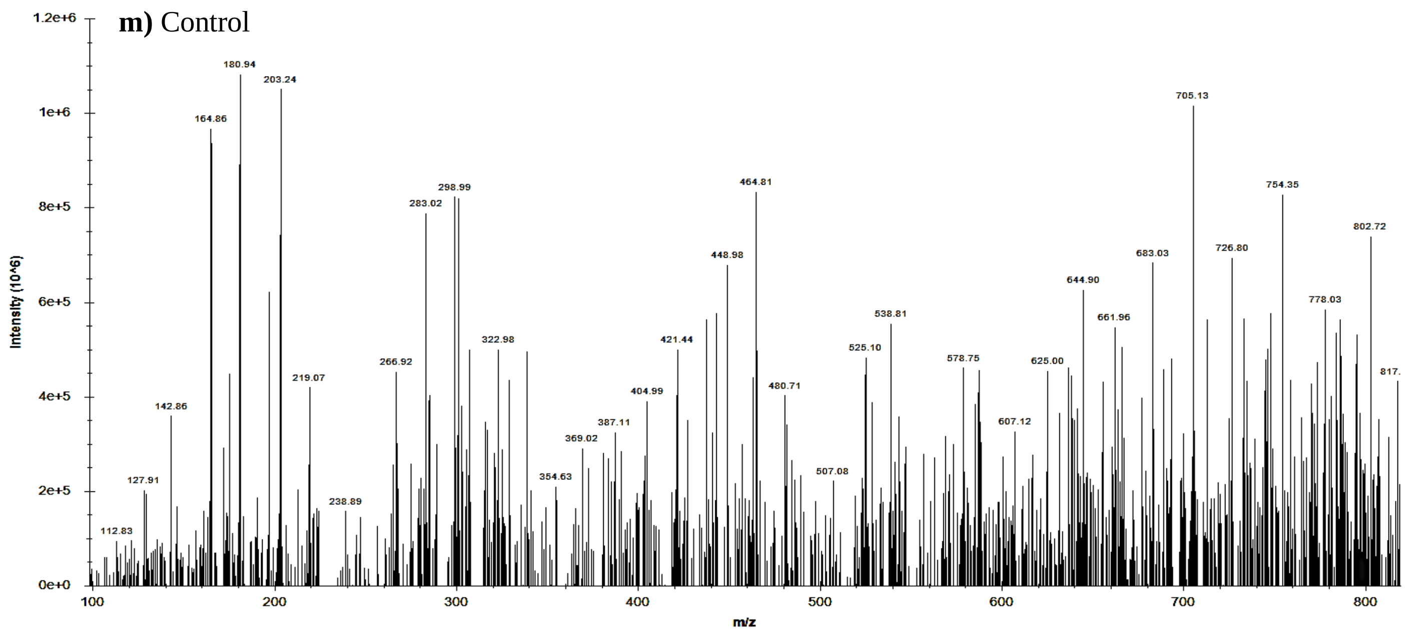

Figure S2 DIESI-MSQD spectra of exo-metabolomes from rhizosphere *Burkholderia* sensu lato strains. The x-axis indicates the *m/z* ratio, whereas the y-axis plots the relative signal intensity of each ion. The images illustrate a representative foot-print of the bacterial supernatants analyzed. The spectra are organized as follows, Clade I: **a)** *B. contaminans* MSR2; **b)** *B. metallica* 1Ac2; **c)** *B. arboris* 1Ac4, and **d)** *B. ubonensis* PEI4; Clade II: **e)** *B. gladioli* 1Ac1; **f)** *B. gladioli* 2Ma15, and **g)** *B. gladioli* 1Ma4; Clade III: **h)** *P. dilworthii* FCV2; **i)** *P. graminis* SCV16; **j)** *P. rhynchosiae* SCV21; **k)** *P. kirstenboschensis* SCV25, and **l)** *T. dinghuensis* 2Ma17. The last image **m)** Control represents the mass spectrum of uninoculated culture media M9.

Table S1. Phylogenetic similarity of bacteria isolated from maize rhizospheric soil.

Isolate	Identity*	Strain with highest phylogenetic similarity	Pairwise similarity (%)
**MSR2**	*B. contaminans*	LMG23361	99.93
**1Ac4**	*B. arboris*	R24201(T)	99.71
**1Ac2**	*B. metallica*	AM747632	99.78
**PEI4**	*B. ubonensis*	CIP-107078	99.31
**1Ac1**	*B. gladioli*	NBRC13700	99.93
**2Ma15**	*B. gladioli*	NBRC13700	99.93
**1Ma4**	*B. gladioli*	NBRC13700	99.71
**SCV16**	*P. graminis*	C4D1M(T)	99.03
**FCV2**	*P. dilworthii*	WSM3556	97.02
**SCV25**	*P. kirstenboschensis*	Kb15(T)	97.76
**SCV21**	*P. rhynchosiae*	WSM3937	97.79
**2Ma17**	*T. dinghuensis*	DHOM06	99.05

* High sequence similarities (> 97%) with the 16S rRNA genes compiled in the EzBioCloud 16S database.

Table S2. Fragmentation ion patterns of selected signals obtained from DIESI-MS analysis of rhizospheric *Burkholderia* sensu lato culture supernatants.

m/z	**Ionization mode**	**MS/MS 10V**	**MS/MS 20V**	**MS/MS 40V**	**MS/MS 50V**	**MS/MS 60eV**	**MS/MS 70eV**	**Putative identity**	**Mol. weight**	**Mol. Formula**	**Precursor type**	**Reference**	**Id. level (A-D)**[‡]
190	Positive	190	190, 162, 120, 71, 45	190, 162, 120, 101, 71, 45	-	-	-	Pyochelin	324.413	$C_{14}H_{16}N_2O_3S_3$	[M-135+H]+		
210	Positive	210	210, 120, 91, 73, 57	210, 178, 120, 118, 91, 73, 57, 45	-	-	-	n.i.	-	-	-		
275	Positive	275	275	275, 137, 123, 112	-	-	-	n.i.	-	-	-		
325	Positive	325, 128	325, 224, 206, 190, 172, 146, **128**, 100, 70	325, 210, **190**, 172, 146, 128, 120, 73, 57, 39	-	-	-	Pyochelin	324.413	$C_{14}H_{16}N_2O_3S_3$	[M+H]+	[53]	A[*1]
347	Positive	347, 246	347, 303, 269, **246**, **190**, 168, 156, 128, 94, 85,23	347, 213, **190**, 163, 142, 120, 94, 71, 23	-	-	-	Pyochelin	324.413	$C_{14}H_{16}N_2O_3S_3$	[M+Na]+	[53]	A
363	Positive	363. 39	39	39	-	-	-	Pyochelin	324.413	$C_{14}H_{16}N_2O_3S_3$	[M+K]+	[53]	A
369	Positive	369, 360	369, 334, 282, 229, 210, 159, 149, **131**, **114**, 97, 89, 72, 55, 41	243, 200, 184, 149, **131**, **114**, 86, 72, 55, 41	-	-	-	Bacteriohopane C35 Skeleton	369.6565	$C_{27}H_{45}$+	M^+	[54]	A
380	Positive	380	380	380, 378, 334, 247, 227, 191, **131**, **114**, 97, 89, 86, 72, 70, 23	-	-	-	n.i.	-	-	-		
391	Positive	390	390	390, 356, 353, 326, 269, 220, 134, 114, 23	-	-	-	n.i.	-	-	[M+Na]+		
393	Positive	393, 335	393, 246, 39	393, 159, 130, 114, 39, 23	-	-	-	n.i.	-	-	[M+Na]+		
409	Positive	-	-	-	409, 404, 353, 282, 265, 262, 178, 138, 122, 39	409, 388, 317, 365, 187, 169, 139, 93, 39	409, 401, 364, 282, 265, 261, 186, 137, 116, 39	n.i.	-	-	-		
410	Positive	-	-	-	410, 380, 346, 267, 239, 179, 141, 39	410, 378, 266, 209, 178, 165, 124, 95, 39	410, 405, 352, 316, 265, 222, 197, 165, 119, 63, 39	n.i.	-	-	-		
475	Positive	-	-	-	475, 23	475, 34	475, 144, 113, 23	n.i.	-	-	-		

476	Positive	-		-	476, 246, 165, 135, 39	476, 389, 268, 73, 23	476, 470, 453, 431, 332, 181, 138, 40, 23	n.i.	-	-	-		
479	Positive	-	-	-	479, 474, 443, 382, 336, 289, 266, 181, 159, 113, 39	479, 475, 461, 396, 333, 305, 363, 182, 165, 39	479, 477, 473, 455, 388, 382, 380, 352, 319, 304, 279, 202, 182, 165, 158, 39, 23	n.i.	-	-	-		
480	Positive	-	-	-	480, 477, 435, 365, 335, 266, 165, 114, 39, 23	480, 475, 453, 402, 336, 253, 237, 193, 141, 42, 39, 23	480, 476, 474, 456, 391, 369, 274, 151, 102, 39, 23	n.i.	-	-	-		
491	Positive	-	-	-	491, 489, 487, 430, 371, 347, 238, 165, 143, 39	491, 489, 487, 430, 376, 349, 178, 151, 88, 39	491, 488, 475, 432, 426, 348, 229, 181, 142, 39, 23	n.i.	-	-	-		
492	Positive	-	-	-	492, 489, 23	492, 489, 429, 357, 348, 158, 142, 44, 39, 23	492, 489, 484, 469, 410, 380, 305, 264, 248, 151, 122, 57, 38, 22	n.i.	-	-	-		
507	Positive	-	-	-	507, 502, 431, 388, 340, 268, 251, 231, 181, 164, 38	507, 501, 426, 379, 322, 265, 229, 122, 38, 23	506, 502, 448, 423, 370, 329, 289, 267, 185, 165, 135, 39, 23	n.i.	-	-	-		
508	Positive	-	-	-	508, 503, 491, 409, 381, 301, 264, 25	508, 503, 409, 360, 312, 278, 209, 165, 135, 39	506, 483, 418, 406, 240, 174, 32, 23	n.i.	-	-	-		
509	Positive	-	-	-	509, 428, 411, 410, 338, 266, 40, 37	509, 479, 429, 411, 389, 252, 162, 143, 113, 41, 39, 36	507, 475, 423, 348, 377, 328, 279, 250, 208, 206, 153, 113, 39, 23	n.i.	-	-	-		
521	Positive	-	-	-	521, 517, 445, 401, 328, 286, 198, 165, 45, 39	521, 517, 514, 456, 407, 356, 288, 183, 177, 165, 38	521, 516, 445, 397, 383, 303, 267, 228, 187, 165, 102, 39, 23	n.i.	-	-	-		
523	Positive	-	-	-	523, 491, 445, 409, 405, 380, 359, 282, 238, 39	523, 490, 459, 420, 411, 363, 288, 263, 165, 39	522, 491, 486, 484, 458, 447, 379, 333, 286, 266, 188, 164, 120, 97, 39	n.i.	-	-	-		
524	Positive	-	-	-	524, 521, 492, 380, 300, 249, 207, 165, 102, 86, 39	523, 492, 460, 394, 289, 181, 165, 39	523, 519, 488, 486, 359, 286, 240, 41	n.i.	-	-	-		

619	Positive	-	-	-	619, 559, 462, 421, 23	619, 608, 527, 463, 383, 23	618, 552, 520, 450, 430, 356, 165, 37	n.i.	-	-	-		
622	Positive	-	-	-	621, 613, 595, 502, 458, 422, 374	622, 602, 585, 401, 214, 192, 112	622, 613, 576, 478, 264, 196, 179, 160, 33	n.i.	-	-	-		
635	Positive	-	-	-	635, 515, 491, 394, 359, 338, 39, 21	635, 522, 491, 393, 265, 181, 149, 39	636, 576, 523, 495, 478, 464, 393, 350, 283, 189, 164, 112, 39	n.i.	-	-	-		
636	Positive	-	-	-	636, 581, 525, 491, 427, 394, 387, 39	635, 595, 491, 393, 368, 283, 39	363, 613, 576, 394, 165, 143, 39	n.i.	-	-	-		
701	Positive	-	-	-	701	701, 568, 319, 213, 20	701, 20	n.i.	-	-	-		
702	Positive	-	-	-	702, 643, 574, 543, 37	702, 523, 28	702, 526, 412, 383, 287, 175, 22	n.i.	-	-	-		
709	Positive	-	-	-	709, 661, 592, 431, 361, 348	709, 693, 641, 607, 571, 502, 469, 384, 321, 175, 158, 113, 39	709, 677, 633, 517, 381, 304, 165, 148, 59,	n.i.	-	-	-		
709	Positive	-	-	-	709, 675, 383, 237, 185, 89	709, 633, 563, **465**, 405, **334**, 283, 245, 222, 200, 130, 114, 89, 86	709, 691, 550, **465**, 405, 365, **334**, 310, 283, 222, 212, 183, 113, 89, 86, 68	Ornibactin C6	708.767	$C_{28}H_{52}N_8O_{13}$	$[M+H]^+$	[55]	B*[2]
721	Positive	-	-	-	721, 333, 89	721, 717, 703, 559, 465, 437, 404, 334, 228, 211, 98, 97, 86, 70	721, 703, 561, 475, 449, 333, 318, 257, 231, 202, 131, 98, 70	Ornibactin C6	708.767	$C_{28}H_{52}N_8O_{13}$	$[M+Na]^+$		C
722	Positive	-	-	-	722	722, 704, 610, 333, 231, 72	251, 236, 231, 159, 131, 98, 70	n.i.	-	-	-		
737	Positive	-	-	-	737, **465**, 334, 247	737, **465**, 334, 247, 131, 89, 70	737, **465**, 334, 247, 131, 89, 86, 70	Ornibactin C8	736.821	$C_{30}H_{56}N_8O_{13}$	$[M+H]^+$	[55]	B*[3]
759	Positive	-	-	-	759, 685, 599, 582, 426, 357, 29	685, 599, 487, 356, 269, 221, 189, 38	685, 556, 487, 425, 356, 330, 269, 175, 39, 23	Ornibactin C8	736.821	$C_{30}H_{56}N_8O_{13}$	$[M+Na(23)]^+$		C
760	Positive	-	-	-	-	760, 742, **356**, 326, 242, 155, 67	760, 742, 686, 560, 586, 516, 426, **356**, 335, 235, 209, 136, 39	Ornibactin C8 (*isotope +1*)	737.821	$C_{30}H_{56}N_8O_{14}$	$[M+Na(23)]^+$		

775	Positive	-	-	-	775, 756, 649, 634, 372, 330, 39, 23	775, 756, 702, 634, 572, 439, 372, 181, 39, 23	775, 756, 731, 702, 634, 580, 442, 372, 222, 39, 23	Ornibactin C8	736.821	$C_{30}H_{56}N_8O_{13}$	$[M+K(39)]^+$		C
780	Positive	-	-	-	781, **455**, 356, 173, 21	-	781, 682, 607, 542, **455**, 443, 387, 335, 291, 205, 161, 91, 39	n.i.	-	-	-		
782	Positive	-	-	-	782, 746, 677, 620, 460	782, 443	782, 456, 441, 388, 264, 219, 39	n.i.	-	-	-		
797	Positive	-	-	-	797, 567, **471**, 403, 375	797, 686, **471**, 404, 39	797, 782, 686, **471**, 247, 146, 39	n.i.	-	-	$[M+K(39)]^+$		
178	Negative	-	178, 118	178, 173, 143, 118	-	-	-	Pyochelin	324.413	$C_{14}H_{16}N_2O_3S_3$	[M-H-148]-		A
204	Negative	204	204, 176, 118	204, 202, 199, 176, 144, 118	-	-	-	Pyochelin	324.413	$C_{14}H_{16}N_2O_3S_3$	[M-H-119]-		A
206	Negative	206, 118	206, 176, 118	206, 176, 161, 118, 108, 91	-	-	-	Pyochelin	324.413	$C_{14}H_{16}N_2O_3S_3$	[M-H-117]-		A
222	Negative	222, 118	222	222, 189, 118	-	-	-	Pyochelin	324.413	$C_{14}H_{16}N_2O_3S_3$	[M-H-101]-		A
245	Negative	245	245, 189, 126, 118	245, 215, 202, 189, 118	-	-	-	Pyochelin	324.413	$C_{14}H_{16}N_2O_3S_3$	[M-H-78]-		A
261	Negative	261, 243	261, 246, 243, 228, 201, 118	261, 259, 243, 206, 134, 118, 79	-	-	-	Pyochelin	324.413	$C_{14}H_{16}N_2O_3S_3$	[M-H-62]-		A
323	Negative	323, 245, 222	323, 245, 222, 178, 118	322, 245, 222, 189, 178, 118	-	-	-	Pyochelin	324.413	$C_{14}H_{16}N_2O_3S_3$	[M-H]-	[53]	A
345	Negative	345	345, 301, 244	345, 300, 244, 183, 163, 138, 118, 89	-	-	-	Pyochelin	324.413	$C_{14}H_{16}N_2O_3S_3$	[M-Na-2H]-		A
609	Negative	609	609	609, 602, 492	-	-	-	n.i.	-	-	-		
647	Negative	647, 323	647, 323	647, 323, 245, 222, 178, 118	-	-	-	Pyochelin	324.413	$C_{14}H_{16}N_2O_3S_3$	[2M-H]-		C
719	Negative	-	719, 630	-	719, 386	-	-	Deoxyornibactin C8	720.821	$C_{30}H_{56}N_8O_{11}$	[M-H]-		C
735	Negative	-	735	735, 700, 470, 427	-	735, 427, 397, 285	-	Ornibactin C8	736.821	$C_{30}H_{56}N_8O_{13}$	[M-H]-	[55]	B

*** Link for database:** [1]mona.fiehnlab.ucdavis.edu/spectra/display/CCMSLIB00005724305; [2]mona.fiehnlab.ucdavis.edu/spectra/display/CCMSLIB00001059072; [3]mona.fiehnlab.ucdavis.edu/spectra/display/CCMSLIB00001059073

‡**Identification Level**: **A**(Standard), **B** (MS/MS + Reference) and **C** (MS/MS)
